# Evolution of a minimal cell

**DOI:** 10.1038/s41586-023-06288-x

**Published:** 2023-07-05

**Authors:** R. Z. Moger-Reischer, J. I. Glass, K. S. Wise, L. Sun, D. M. C. Bittencourt, B. K. Lehmkuhl, D. R. Schoolmaster, M. Lynch, J. T. Lennon

**Affiliations:** 1 Department of Biology, Indiana University, Bloomington, IN USA; 2 J. Craig Venter Institute, La Jolla, CA USA; 3 Novartis Gene Therapy, San Diego, CA USA; 4 Embrapa Genetic Resources and Biotechnology, National Institute of Science and Technology in Synthetic Biology, Brasília, Brazil; 5 US Geological Survey, Wetland and Aquatic Research Center, Lafayette, LA USA; 6 Arizona State University, Tempe, AZ USA

**Keywords:** Experimental evolution, Bacterial genes, Genome evolution, Synthetic organisms, Molecular evolution

## Abstract

Possessing only essential genes, a minimal cell can reveal mechanisms and processes that are critical for the persistence and stability of life^1,2^. Here we report on how an engineered minimal cell^3,4^ contends with the forces of evolution compared with the *Mycoplasma mycoides* non-minimal cell from which it was synthetically derived. Mutation rates were the highest among all reported bacteria, but were not affected by genome minimization. Genome streamlining was costly, leading to a decrease in fitness of greater than 50%, but this deficit was regained during 2,000 generations of evolution. Despite selection acting on distinct genetic targets, increases in the maximum growth rate of the synthetic cells were comparable. Moreover, when performance was assessed by relative fitness, the minimal cell evolved 39% faster than the non-minimal cell. The only apparent constraint involved the evolution of cell size. The size of the non-minimal cell increased by 80%, whereas the minimal cell remained the same. This pattern reflected epistatic effects of mutations in *ftsZ*, which encodes a tubulin-homologue protein that regulates cell division and morphology^5,6^. Our findings demonstrate that natural selection can rapidly increase the fitness of one of the simplest autonomously growing organisms. Understanding how species with small genomes overcome evolutionary challenges provides critical insights into the persistence of host-associated endosymbionts, the stability of streamlined chassis for biotechnology and the targeted refinement of synthetically engineered cells^2,7–9^.

## Abstract (web summary)

An engineered minimal cell evolves to escape the negative consequences of genome streamlining.

## Main

The complexity of a genome is reflected by the number of genes that it contains, a quantity that varies by orders of magnitude across the tree of life. Whereas some obligately endosymbiotic bacteria have fewer than 200 protein-coding genes, many plant and animal genomes contain more than 20,000 genes^10–12^. In principle, the simplest organism is one that possesses only the minimum number of genes for survival and reproduction in a given environment. Any mutation in such an organism could lethally disrupt one or more cellular functions, placing constraints on evolution, as revealed by the fact that essential proteins change more slowly than those encoded by dispensable genes^13,14^. Furthermore, organisms with streamlined genomes have fewer targets on which positive selection can act, therefore limiting opportunities for adaptation.

The cell is the simplest independent functional unit of life. However, even unicellular model organisms that are touted for their tractability are complex, possessing thousands of genes and proteins, many of which remain uncharacterized even after decades of in-depth investigation. The quest for the simplest organism has been aided by advances in synthetic biology, which involves the redesign or novel construction of biological parts and modules^2,15^. Synthetic biology provides a platform for developing powerful simplest-case models through streamlining, whereby non-essential sequences are removed from an organism’s genome^1–3,8,16^. Guided by such strategies, a minimal cell was constructed with a genome containing only the smallest set of genes required for autonomous cellular life^3,4^. Although these efforts succeeded in experimentally identifying the genetic requirements for basic cellular processes, such as metabolism and cell division, it remains unclear how a minimal cell will respond to the forces of evolution. On one hand, evolution of a minimal cell could be constrained by the limited raw materials with which natural selection can operate. On the other hand, synthetic streamlining may result in a highly disrupted genome, altering protein interactions and expanding the opportunity for adaption to a new cellular environment.

To gain insights into the dynamics and outcomes of evolution in a minimal cell, we conducted experiments with strains of *M. mycoides*^3,4^, which are bacteria belonging to the Mollicutes. The minimal cell (JCVI-syn3B) has a synthetically constructed genome containing a subset of genes found in a corresponding non-minimal strain (JCVI-syn1.0). By reducing the chromosome from 901 to 493 genes, JCVI-syn3B has the smallest genome of any organism that can be grown in pure laboratory culture^3,4^. With these two strains, we first investigated whether genome streamlining—which included the removal of two DNA-replication genes, eight DNA repair genes and other genes of unknown function—altered the rate and spectrum of new mutations in the minimal cell relative to the non-minimal organism under conditions of relaxed selection. Second, with knowledge of the mutational input, we evaluated whether genome minimization altered the rate and mechanisms of evolution in response to natural selection, as measured using whole-genome sequencing, estimates of population fitness and phenotypic changes in cell size.

## Highest recorded mutation rate

**Fig. 1:**
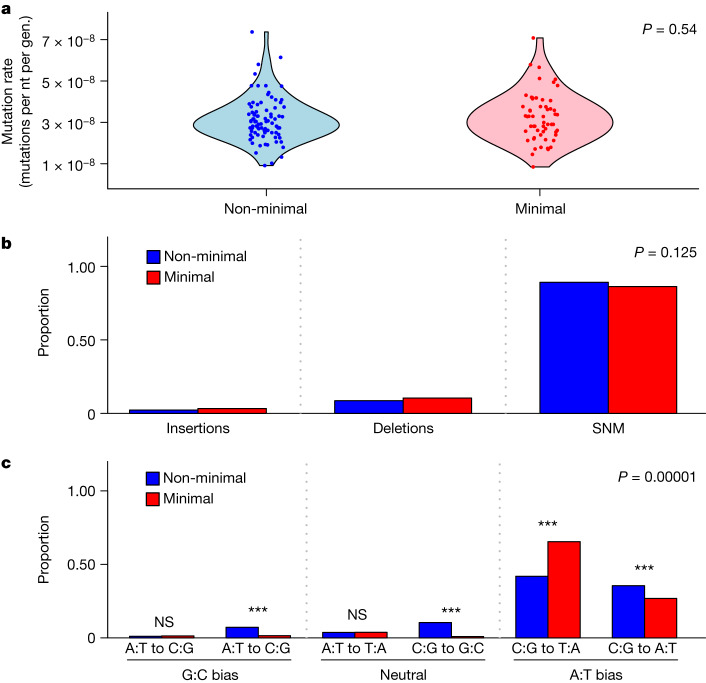
The mutation rate and spectrum of the minimal and non-minimal cell. **a**–**c**, The mutation rate (per nucleotide (nt) per generation (gen.)) and spectrum of the minimal and non-minimal cell were estimated from mutation-accumulation experiments. **a**, Although synthetic *M. mycoides* has the highest recorded mutation rate (base substitutions and indels), it was not affected by genome minimization. The dark coloured circles represent non-minimal (*n* = 85) and minimal (*n* = 57) clones that were sequenced at the end of the experiment. The light coloured areas represent kernel densities of the data. **b**, The proportions of insertions, deletions and SNMs were also the same for the minimal and non-minimal cells. **c**, Among SNMs, which accounted for 88% of all mutations, the minimal cell exhibited a stronger A:T bias in its mutation spectrum compared with the non-minimal cell, particularly in the C:G to T:A category. Two-sided *χ*^2^ analysis was used for hypothesis testing; ***P = 2.5 × 10^−6^ (A:T to G:C), ***P = 1.5 × 10^−11^ (C:G to G:C), ***P = 1.6 × 10^−20^ (C:G to T:A), ***P = 0.0003 (C:G to A:T); NS, not significant. Source Data

Through serial bottlenecking under relaxed selection, we conducted mutation accumulation experiments with populations of *M. mycoides* (Methods). The number of mutations per nucleotide per generation for the non-minimal cell (3.13 ± 0.12 × 10^−8^, mean ± s.e.m.) was indistinguishable from that of the minimal cell (3.25 ± 0.16 × 10^−8^) (*t*_140_ = 0.43, *P* = 0.667; Fig. 1a). These mutation rates, which are the highest recorded for any cellular organism, are consistent with other reports in which organisms with smaller genomes have higher mutation rates^17–20^. Notably, the mutation rate was not affected by genome minimization that included the elimination of genes involved in replication fidelity (Fig. 1a). Perhaps this is due to the fact that *M. mycoides* already has an elevated mutation rate. To evaluate the generality of our findings, the effect of genome minimization should be investigated in a microorganism with a lower intrinsic mutation rate. In any case, our data are consistent with predictions from the drift-barrier hypothesis. This theory posits that mutation rates evolve downwards until the selective advantage of another incremental decrease in the mutation rate is small enough to be effectively neutral and outweighed by genetic drift^19,20^. In other words, populations with a lower effective population size (*N*_e_) experience stronger drift and, therefore, evolve higher mutation rates^19^. Notably, wild-type *M. mycoides* is an obligate pathogen and has genomic features (small genome size and low GC content) consistent with it having a low *N*_e_^17,18,21,22^. Note that mutation-accumulation studies are typically designed to estimate the rate and spectrum of viable mutations. By eliminating redundancy, genome streamlining could alter the contribution of strongly deleterious or lethal mutations that would not be captured in our study.

## Minimization and mutational spectrum

Although the mutation rate was robust to genome streamlining, the types of mutations that arise in a population can still influence evolution. Overall, the composition of mutation types (insertions, deletions and single-nucleotide mutations (SNMs)) was not affected by genome minimization (*χ*^2^_2_ = 4.16, *P* = 0.125; Fig. 1b). However, the composition of SNMs, which constituted the largest category of mutations (88%), differed between the minimal and non-minimal cells (Monte Carlo *χ*^2^ = 69.9, *P* = 1.0 × 10^−4^). For both cell types, mutations from a G or C nucleotide to an A or T nucleotide occurred at a higher rate compared with mutations in the opposite direction, that is, from A or T to G or C (Fig. 1c; non-minimal cell, *χ*^2^_1_ = 3736, *P* < 2.2 × 10^−16^; minimal cell, *χ*^2^_1_ = 1444, *P* < 2.2 × 10^−16^). The magnitude of this A:T bias was affected by genome streamlining (*χ*^2^_1_ = 21.8, *P* = 3.08 × 10^−6^; Fig. 1c) leading to a 30-fold bias in the non-minimal cells and a 100-fold bias in the minimal cells. The discrepancy is probably due to the deletion of *ung*, a gene of which the protein product excises misincorporated uracil that can otherwise cause C-to-T mutations^23^. Its removal from the minimal cell’s genome should elevate A:T mutational bias relative to the non-minimal cell as observed.

## Recovery of fitness in a minimal cell

With mutation rates of around 3 × 10^−8^ per nucleotide per generation and population sizes in excess of 10^7^ individuals, a new mutation would hit every nucleotide in the genome more than 250 times during 2,000 generations of experimental evolution. Thus, neither cell type would be limited by the availability of genetic variation to fuel adaptation. Any differences in the ways the two strains adapt should be driven by alterations in genome content created by synthetic streamlining.

**Fig. 2:**
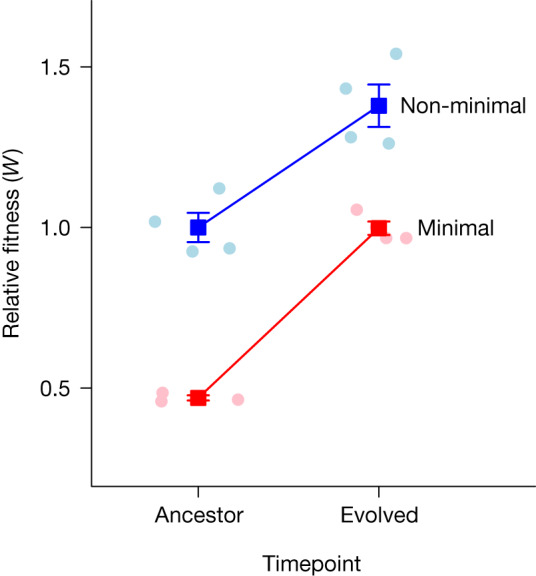
The effect of genome minimization on fitness and adaptation. Genome minimization reduced the relative fitness by 50%. However, almost all of this cost was regained over 2,000 generations of evolution. Despite the removal of nearly half of its genome, the minimal cell adapted at a rate comparable to that of the non-minimal cell, which was corroborated by fitness estimates from growth curve experiments (Extended Data Fig. 1 and Extended Data Table 1). The dark coloured symbols represent mean ± s.e.m. As the experiment was initiated with a single clone, error bars for the ancestral timepoint were calculated from technical replicates (*n* = 4), whereas error bars for evolved populations were calculated from replicate populations (*n* = 4), both of which are depicted by light coloured symbols. The solid red and blue lines are a visual aid connecting the mean values of the minimal and non-minimal populations, respectively. Source Data

**Extended Data Fig. 1:**
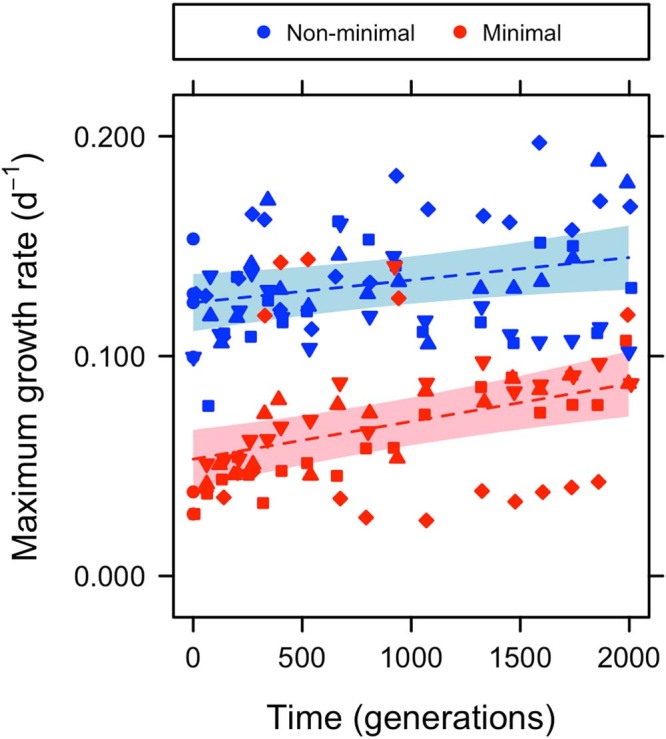
Trajectories of maximum growth rates (*µ*max) for the minimal cell and non-minimal cell. Data (*n* = 141) were generated from growth-curve assays that were fit using a modified Gompertz equation (see Fig. S5) across 2000 generations of experimental evolution. With these estimates of *µ*max, we then fit a generalized linear mixed model (GLMM) where time (generation) and cell type (minimal cell vs. non-minimal cell) were treated as fixed effects and replicate evolved populations (n = 8) was treated as a random effect. Based on the intercepts from the GLMM, synthetic streamlining reduced *µ*max by 57% in the non-evolved ancestors. During subsequent evolution, *µ*max for both cell types increased at comparable rates over the course of the experiment (see Extended Data Table 1). In the figure, dark-coloured circles represent data from the ancestral populations, while triangles (up- and down-pointing), diamonds, and squares represent data from the replicate evolved populations. Dashed lines and light-coloured regions represent predicted values and 95% confidence intervals, respectively, for the fixed effects (generation and cell type). The conditional R^2^, which accounts for variance explained by the fixed and random effects, was 0.68. The variance partition coefficient (VPC) of 0.127 indicates that an appreciable portion of the total explained variance in *µ*max was associated with the random effect of the replicate evolved populations (See Extended Data Table 1). Additional information, including model fits, parameters, summary statistics, and residual plots, can be found in the online Figshare repository. Source Data

To study natural selection, we passaged replicate populations of *M. mycoides* for 2,000 generations (Methods), a period during which rapid adaptation is often observed^24,25^. We then measured fitness, the contribution of a genotype’s offspring to future generations, using two methods^26^. First, we quantified the maximum growth rate (*µ*_max_) of each replicate population every 65–130 generations (Methods). We documented that genome streamlining led to a 57% reduction in *µ*_max_, but that this measure of fitness subsequently increased linearly and at comparable rates for the minimal cell (1.71 × 10^−5^ ± 4.53 × 10^−6^ per day per generation) and non-minimal cell (1.03 × 10^−5^ ± 4.53 × 10^−6^ per day per generation) during the evolution experiment. Using the predicted values from a generalized linear mixed model, the *µ*_max_ of the non-minimal and minimal cell increased by 17–68% over the course of the experiment (Extended Data Fig. 1 and Extended Data Table 1). Second, we measured relative fitness using head-to-head competition assays with the ancestral (generation 0) and most evolved (generation 2,000) populations (Methods). For the ancestral strains, we determined that genome minimization led to a 53% decrease in fitness (Fig. 2), on par with estimates based on *µ*_max_. Despite this major initial cost, the minimal cell rapidly regained fitness. In fact, the competition-based estimates of fitness indicate that the minimal cell adapted 39% more rapidly than the non-minimal cell (*t* = −2.530, *P* = 0.032). With the power afforded by our experimental design, the average relative fitness of the evolved minimal cell (0.998) was statistically indistinguishable (*t* = −0.055, *P* = 0.957) from that of the ancestral non-minimal cell (1.00). Given this, we conclude that effectively all of the fitness lost to genome streamlining was recovered during 300 days of serial passaging (Fig. 2 and Supplementary Fig. 1). Our findings suggest that a streamlined *M. mycoides* genome is not inherently crippled and can perform as well as the non-minimized cell after readaptation.

**Extended Data Fig. 2:**
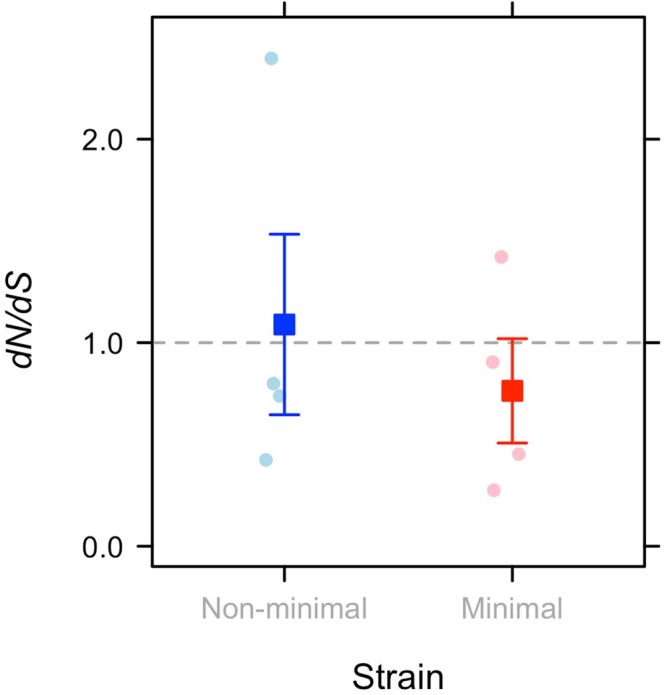
Effect of genome streamlining on the ratio of nonsynonymous to synonymous substitutions. In populations of *Mycoplasma mycoides* after 2000 generations of evolution, we used the normalized ratio of nonsynonymous to synonymous mutations (*d*_*N*_/*d*_*S*_) as an indicator of natural selection. Values of *d*_*N*_/*d*_*S*_ > 1 are associated with positive selection, while values of *d*_*N*_/*d*_*S*_ < 1 are associated with the dominance of negative selection and constraint on adaptation. The minimal and non-minimal cell exhibited comparable values of *d*_*N*_/*d*_*S*_ (*t*_6_ = 0.81, *P* = 0.488). One of the replicate populations belonging to the non-minimal treatment had an elevated *d*_*N*_/*d*_*S*_ (2.06) compared to other replicate populations (mean *d*_*N*_/*d*_*S*_ = 0.45). When we removed this potential outlier, there was still no difference in *d*_*N*_/*d*_*S*_ between the minimal and non-minimal cell (*t*_5_ = −0.25, *P* = 0.811). Dark-coloured symbols represent the mean ± SEM (*n* = 4). Light-coloured symbols represent individual values for each replicate population (*n* = 4). Hypotheses were evaluated with two-sided *t*-tests. Source Data

On the basis of the fitness dynamics, we conclude that adaptation was not constrained by genome minimization. This interpretation was bolstered by results from population genomic sequencing (Methods). The relative ratio of nonsynonymous to synonymous fixed SNMs (*d*_N_/*d*_S_) was similar between the two cell types (*t*_6_ = 0.81, *P* = 0.488; Extended Data Fig. 2), consistent with the interpretation that the rates of molecular evolution were comparable even though almost all of the genes in the minimal cell are critical for fitness^13,14^.

## Divergent mechanisms of adaptation

**Fig. 3:**
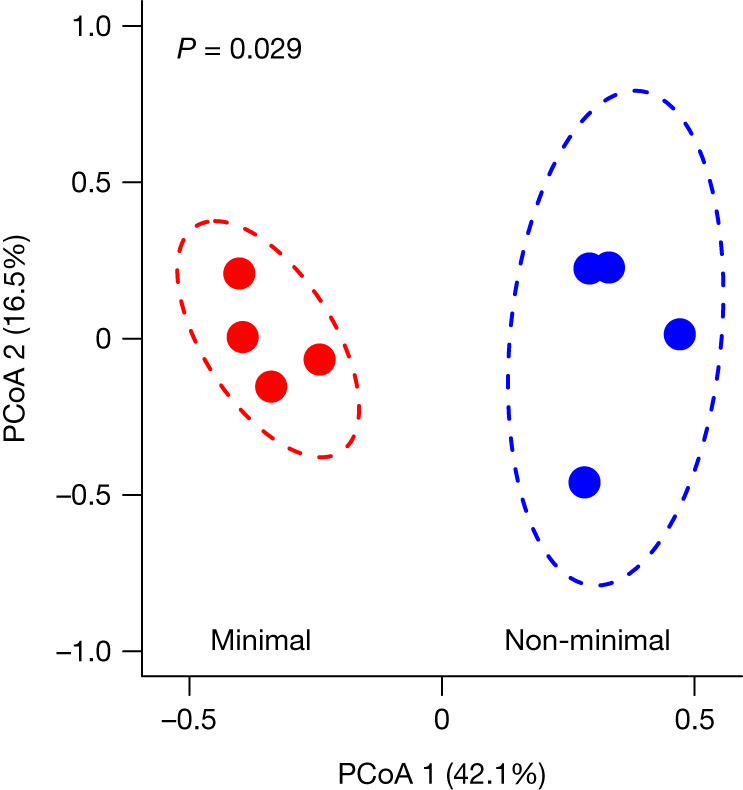
The non-minimal cell and minimal cell populations acquired adaptive mutations in different sets of shared genes. Ordination from a principal coordinates analysis (PCoA) created by a gene-by-population matrix using the Bray–Curtis distance metric after 2,000 generations of evolution (Extended Data Tables 2–4). The dashed lines represent 95% confidence ellipses around replicate populations (*n* = 4 for each cell type) represented by dark coloured symbols. Source Data

**Extended Data Fig. 3:**
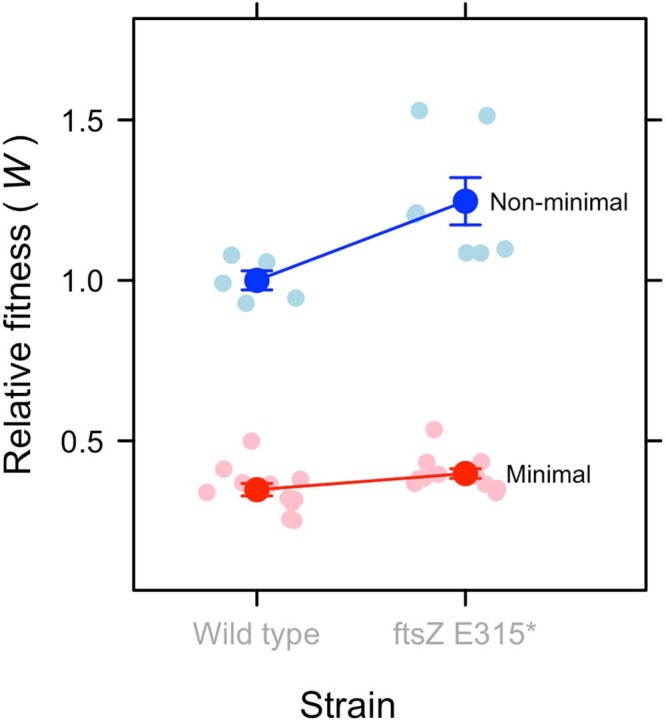
Fitness effects of an *ftsZ* mutation on populations of *Mycoplasma mycoides*. We reengineered the nonsense mutation *ftsZ* E315* and quantified its effect on relative fitness in both the non-minimal and minimal cells using head-to-head competition assays. The *ftsZ* E315* nonsense mutation had a significant effect on *Mycoplasma* cell size that depended on cell type (two-way ANOVA, *F*_1,32_ = 7.45, *P* = 0.010). Compared to the wild type (non-evolved ancestor), the mutation increased relative fitness by 25% in the non-minimal cell and 14% in the minimal cell. Dark-coloured symbols represent the mean ± SEM. Light-coloured symbols represent values for each replicate population. Samples sizes are as follows: wild-type minimal cell, *n* = 12; *ftsZ* E315* minimal, *n* = 12; wild-type non-minimal cell, *n* = 5; *ftsZ* E315* non-minimal, *n* = 5. Source Data

Using a combination of statistical simulation and reverse genetics, we identified mutations that probably contributed to the observed patterns of adaptation. First, we analysed the gene-by-population matrix for nonsynonymous mutations that arose in the shared set of essential genes during the natural selection experiment (Methods). The two cell types acquired mutations in different sets of essential genes (permutational multivariate analysis of variance (PERMANOVA), *F*_7_ = 4.12, *P* = 0.029; Fig. 3) suggesting that the populations evolved through divergent routes. To examine this hypothesis, we looked for genes that acquired a higher number of nonsynonymous, nonsense and small insertion–deletion (indel) mutations than expected under assumptions of neutrality (Methods). We identified 16 genes in the non-minimal genome and 14 in the minimal genome that were potential targets of positive selection (Extended Data Tables 2–4). Second, we used reverse genetics to experimentally verify that one of the common types of mutation observed in replicate populations of both strains was in fact beneficial (Extended Data Table 5). Using CRISPR editing, we recreated *ftsZ* C-terminal nonsense mutations by inserting an *ftsZ* E315* nonsense mutation into the ancestral genomes of the minimized and non-minimized strains (Methods). Head-to-head competition assays with the constructs revealed that this putatively adaptive mutation had a significant effect on *Mycoplasma* performance that was dependent on genome minimization (two-way analysis of variance (ANOVA), *F*_1,32_ = 7.45, *P* = 0.010). The mutation conferred a 25% fitness advantage in the non-minimal cell and a 14% advantage in the minimal cell (Extended Data Fig. 3).

Comparative analysis of the genes putatively under positive selection provided insights into the functional consequences of adaptation in the minimal cell. We hypothesized that mutations in genes related to membrane transport would be critical for adaptation because the minimal cell relies on the import and export of metabolites and other biomolecules for metabolism^4,27^. However, mutations in membrane transport functions were enriched to a similar degree in both cell types (Fisher’s exact test, *P* = 0.934). Instead, we detected a marginal signal of enrichment for mutations in biosynthetic genes for the minimal cell (Fisher’s exact test, *P* = 0.090), including those involved in lipid metabolism. Specifically, *fakA* and *clsA* (Extended Data Table 3) are considered to be essential for synthesizing cardiolipin and other lipids from free fatty acids^4^, which are important for the construction of cell membranes and the regulation of cell division. The gene *lgt* is also critical for membrane construction, encoding the protein that transfers diacylglyceryl moieties to anchor surface lipoproteins in the lipid bilayer^4^. Thus, metabolic innovations involving lipid synthesis and distribution may be more important for the minimal cell than enhanced acquisition of metabolites that are already present in the growth medium.

To better understand the pattern of evolutionary divergence, we compared mutations that arose in essential and non-essential genes over 2,000 generations specifically within the non-minimal cell. After accounting for the relative numbers of essential and non-essential genes, there was no difference in the number of mutations observed between these two genomic partitions (*t*_3_ = 0.646, *P* = 0.565; Supplementary Table 1). Nor was there any measurable difference in *d*_N_/*d*_S_ between essential and non-essential genes (*t*_3_ = 0.91, *P* = 0.423; Supplementary Fig. 2). Among the genes putatively under positive selection, there was no evidence for bias towards either essential or non-essential genes (*χ*^2^_1_ = 0.377, *P* = 0.539; Extended Data Table 2). We identified 11 deletions in the non-minimal cell, ten of which were at non-essential loci (Supplementary Table 2). Most of these were small (1–3 bp) but three deletions were large (1,483, 1,495 and 7,047 bp). In summary, it appears that essential genes did not disproportionately contribute to the molecular of evolution of the non-minimal cell, although we cannot rule out that epistatic interactions between essential and non-essential genes contributed to new cell phenotypes.

## Constraints on the evolution of cell size

**Fig. 4:**
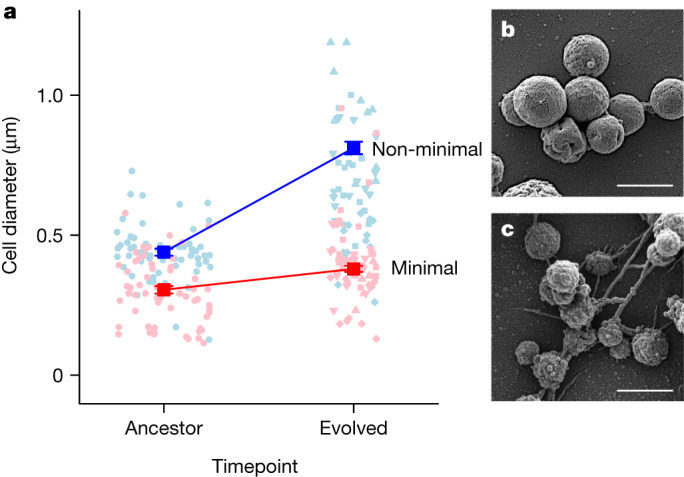
The effect of genome minimization on the evolution of cell size. **a**, Genome minimization was accompanied by a 31% decrease in cell size. Over 2,000 generations of evolution, the size of the non-minimal cells increased by 85% (*P* = 0.005), whereas the size of the minimal cells remained the same (*P* = 0.181). Owing to variation associated with replicate evolved populations, there was a marginal effect when directly comparing changes in the size of the minimal and non-minimal cells (*P* = 0.077; Supplementary Fig. 4). The dark coloured symbols represent the mean ± s.e.m. As the experiment was initiated with a single clone, error bars for the ancestral timepoint were calculated from samples of individuals (*n* = 62 and *n* = 75 for the non-minimal and minimal cell, respectively), whereas error bars at the evolved time point were calculated from individuals (*n* = 285 and *n* = 181 for the non-minimal and minimal cell, respectively) across replicate populations (*n* = 4). The light coloured circles represent randomly drawn data (*n* = 60) corresponding to the diameter of individual cells from the ancestral populations. The light coloured triangles (pointing up and down), diamonds and squares represent randomly drawn data (*n* = 60) corresponding to the diameter of individual cells from the four replicate evolved populations. The solid red and blue lines are a visual aid connecting the mean values of the minimal and non-minimal populations, respectively. **b**,**c**, Scanning electron micrographs obtained from evolved replicate populations of the non-minimal (**b**) and minimal (**c**) cells. Scale bars, 1 μm. Source Data

**Extended Data Fig. 4:**
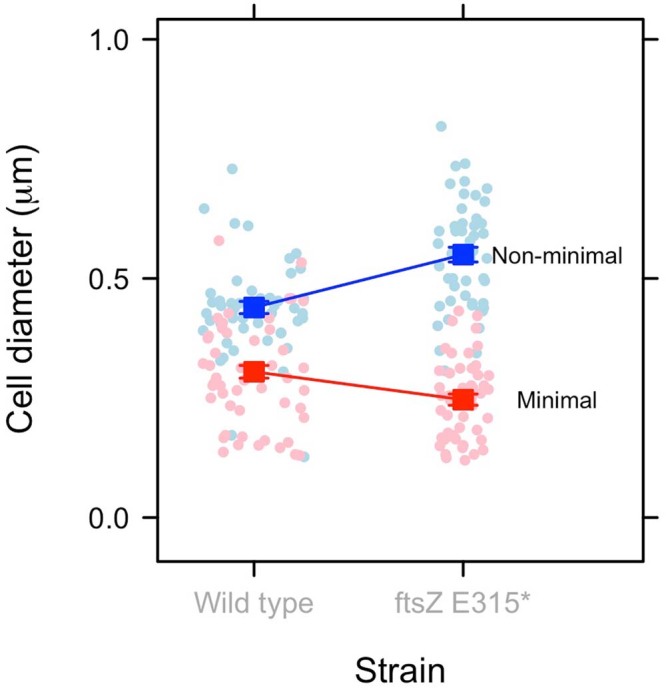
Cell size of *ftsZ* mutants compared to wildtype (non-evolved) for the minimal cell and non-minimal cell. Using scanning electron microscopy, the *ftsZ* E315* nonsense mutation had a significant effect on *Mycoplasma* cell size that depended on cell type (two-way ANOVA, *F*_1,241_ = 37.9, *P* < 0.0001). The mutation in the non-minimal cell caused a 25% increase in cell diameter (*P* < 0.0001) and a corresponding two-fold increase in cell volume. In contrast, the same *ftsZ* nonsense mutation in the minimal cell led to a 19% decrease in the cell diameter (*P* = 0.015). Dark-coloured symbols represent mean ± SEM. Light-coloured symbols represent randomly drawn data (*n* = 60) corresponding to the diameter of individual cells. Source Data

The size of single-celled organisms is variable and often linked to fitness in complex ways^28–30^. In resource-rich environments, cell size tends to be positively correlated with growth rate, one of the most important components of fitness^24,29–32^. For example, in the first 2,000 generations of a classic long-term evolution experiment with *Escherichia coli*, cell volume and fitness concomitantly increased by 50% and 30%, respectively^24^. Although an increase in size can accommodate more macromolecules needed for growth and division, it also decreases a cell’s surface-to-volume ratio, which reduces the efficiency of substrate diffusion. Given these opposing pressures, we evaluated how cell size changed in replicate populations over the course of evolution. Using scanning electron microscopy, we showed that genome streamlining reduced the cell diameter by 31% from 439 ± 0.01 nm to 305 ± 0.01 nm in the ancestral cell types. After 2,000 generation of evolution, the size of the non-minimal cell increased by 85% to 811 ± 0.02 nm (*t* = 3.77, *P* = 0.005), which was accompanied by a tenfold increase in volume compared with its ancestor (Fig. 4 and Extended Data Table 6). By contrast, the size of the minimal cell did not appreciably change (0.08 ± 0.05 nm) during evolution (*t* = 1.51, *P* = 0.181; Extended Data Fig. 4 and Supplementary Fig. 3).

While cell size is a complex multigenic trait, previous studies have attributed changes in morphology of the minimal cell to FtsZ^33^. This protein localizes to the midcell and determines the site of membrane constriction during cell division. Prevalent among diverse lineages of bacteria and archaea^5,6^, *ftsZ* is nevertheless non-essential in *M. mycoides*. However, cells lacking *ftsZ* exhibit aberrant cell division and morphology^3,4,33,34^. Thus, along with 18 other non-essential genes, *ftsZ* was retained in JCVI-syn3B to aid in culture maintenance and stable growth^4,33^. In our study, *ftsZ* was consistently mutated over 2,000 generations of evolution and was identified as a target of positive selection in both the minimal and non-minimal cells (Extended Data Table 4 and Extended Data Fig. 3). Introduction of an early termination codon, as was observed in multiple evolved populations, could eliminate the C-terminal region of the protein that is known to interact with membrane-associated products that recruit FtsZ^35^. The early stop codon could also create a transcriptional polar effect^36^ that reduces expression of two adjacent downstream genes within a probable polycistronic operon—*MMSYN1_0521*, an orthologue of cell division protein *sepF* and *MMSYN1_0520*, encoding aminopeptidase/esterase/lipase, of the α/β hydrolase superfamily^37^. Irrespective of mechanism, we demonstrated that mutations in *ftsZ* had a non-additive effect that contributed to the evolutionary divergence of cell size. We documented that the *ftsZ* E315* nonsense mutation had a significant effect on *Mycoplasma* cell size that was dependent on genome minimization (two-way ANOVA, *F*_1,241_ = 37.9, *P* = 3.1 × 10^−9^). The mutation in the non-minimal cell led to a 25% increase in cell diameter (*P* = 2.0 × 10^−7^) and a corresponding twofold increase in cell volume. By contrast, the same *ftsZ* nonsense mutation in the minimal cell led to a 19% decrease in the cell diameter (*P* = 0.015; Extended Data Fig. 4), which reduced cell volume by half. Thus, the *ftsZ* E315* mutation recapitulated nearly 60% of the evolved divergence in cell size, indicating that FtsZ has a central role in the cell size of *M. mycoides*.

Although changes in *ftsZ* had opposing effects on the size of the minimal and non-minimal cell, mutations in this gene were beneficial for both strains (Extended Data Fig. 3 and Extended Data Table 4). One adaptive consideration is that the faster-growing non-minimal cell should experience bouts of feast-or-famine conditions. In a serial batch environment, repeated transitions between exponential and stationary growth phases has been shown to select for increased cell size^24,28^. The observed patterns may also reflect constraints imposed by genome streamlining on the ability of the minimal cell to evolve an adaptive increase in cell size^29,30,38,39^. With more than 50% of its membrane-transport proteins removed, the minimal cell may have been unable to sequester the resources needed for constructing and maintaining a larger cell^3,30,39^ under the experimental conditions. Alternatively, cell size could evolve as a fitness-neutral byproduct of selection on other traits, such as DNA-replication rate^40^. For example, the two strains could have evolved different size trajectories despite similar selection pressures, due to epistatic effects of genome minimization such as those demonstrated using the *ftsZ*^E315*^ mutants (Extended Data Table 6 and Extended Data Fig. 4). In any case, our findings highlight that cell size—a fundamental feature of biological complexity in multicellular and single-celled organisms alike—evolves in a manner that is dependent on the genomic context.

## Outlook

We uncovered genes, proteins and traits that are critical for evolutionary performance in the synthetically constructed *M. mycoides* JCVI-syn3B—a bacterium with the smallest genome of any organism grown in pure culture in the laboratory. In its ancestral state, this working approximation of a minimal cell had significantly reduced fitness. With less than 500 protein-coding genes, *M. mycoides* JCVI-syn3B had few redundancies when faced with an exceptionally high input of mutations. Despite these challenges, genome reduction did not alter cellular resources in any fundamental way that interfered with the ability to evolve increased fitness. Instead, natural selection during extended laboratory growth outweighed any deleterious effects of genome disruption and drift associated with synthetic streamlining that could have led populations of the minimal cell to extinction.

Our results demonstrate how synthetic biology and engineering can be informed by principles of evolutionary biology and population genetics. While it is now possible to build genomes with desired phenotypes, evolutionary processes represent a powerful but still underdeveloped approach for biological refinement. For example, rapid adaptation of the minimal cell involved selection on distinct targets, 25% of which encoded proteins of unknown function. Future studies combining evolution with a synthetic biology toolset have the potential to improve gene characterization and the mapping of regulatory networks, which may ultimately be used for optimizing stable living systems. Some degree of genome minimization will probably be a common path of development in biotechnology. It would be undesirable if such an approach compromised replication or repair fidelity, owing for example to unexpected cellular changes that might be mutagenic or otherwise interfere with damage maintenance. From an engineering perspective, more studies are needed to evaluate the minimization of other genomes in alternate chassis under different environmental conditions. Nevertheless, if we assume that our findings are somewhat general, it appears that cellular functions are robust to streamlining over time, which is desirable when using minimized cells for biotechnology and bioproduction.

Our findings shed new light on the phenomenon of genome streamlining, which is prevalent in nature, especially among microorganisms that coevolve with hosts in both pathogenic and mutualistic ways, but also among free-living bacteria that dominate the global oceans^7,9,41^. Both adaptive and neutral theories have been developed to explain why genomes become streamlined^42,43^. Very few studies have mechanistically investigated how genome streamlining affects subsequent evolution, especially for microorganisms with different phylogenetic backgrounds living in environments with contrasting niches. Despite it reducing the sequence space of possible trajectories, we conclude that streamlining does not constrain fitness evolution and diversification of populations over time. Genome minimization may even create opportunities for evolutionary exploitation of essential genes, which are commonly observed to evolve more slowly^13,44^.

## Methods

### Strains and growth conditions

We maintained synthetic *M. mycoides* JCVI-syn1.0 and synthetic *M. mycoides* JCVI-syn3B in SP4 medium with KnockOut Serum Replacement (Gibco) substituted for fetal bovine serum (Supplementary Table 3). Cultures of these non-motile bacteria were grown in a dark, static growth chamber at 37 °C. The non-minimal JCVI-syn1.0 strain has been described in detail previously^45^. The minimal JVCI-syn3B is identical to the strain synthesized in previous studies^3^ with the following exceptions: JVCI-syn3B possesses a second rRNA operon copy, lacks a gene (*MMSYN1_0531*) encoding an efflux protein, and has 19 genes that were added back into the minimal genome to render the cell easier to use^4,33^ (Supplementary Table 4). The strain also contains a landing pad system (*cre* recombinase and *loxP*) facilitating genetic manipulation. For competition experiments used to quantify relative fitness, we used a JCVI-syn1.0 strain that expresses mCherry, which enabled us to distinguish it in mixed culture from other strains using flow cytometry and also factor out any costs associated with production of the fluorescent protein (see below).

### Mutation accumulation experiment

#### Overview

Mutation accumulation (MA) experiments are designed to reduce the influence of natural selection through repeated bottlenecks of evolving populations^19^. When used with microbial populations, this is typically achieved by transferring single colonies, which have undergone single-cell bottlenecks. Before initiating MA experiments, we acclimatized JCVI-syn1.0 and JCVI-syn3B to laboratory conditions by maintaining populations in SP4 liquid medium. We took a clone of each acclimated strain to begin the MA experiment and propagated replicate lineages (*n* = 87 and *n* = 57 for JCVI-syn1.0 and JCVI-syn3B, respectively) for 20 to 36 weekly transfers.

#### Number of generations

To compare rates of mutation across replicates, we normalized all rates as per-generation values. To calculate the number of generations per transfer in the MA, we grew cells on SP4 agar for 1 week and diluted a sample of seventh day colonies into 1 ml of phosphate-buffered saline (pH 7.4). Cells were fixed with 20 μl of 25% glutaraldehyde and stained with 2× SYBR Green, and then counted with a NovoCyte flow cytometer (ACEA Biosciences). We used the dilutions to calculate the number of cells in the original colony, from which we inferred the number of generations (log_2_[*N*], where *N* is the number of cells in the undiluted colony) that must have occurred to reach a colony of that size^46^, assuming each colony is formed by a single progenitor cell. As the growth rate and other fitness components can decrease during an MA experiment^47^, we also measured the number of cells per colony during and at the end of the MA, averaging across timepoints to estimate the total number of generations. We then used the number of generations per transfer to estimate the effective population size (*N*_e_) using the harmonic mean method^47^. Specifically, *N*_e_ was approximated as the harmonic mean of the series (2^0^, 2^1^, 2^2^, …, 2^*f*^), where *f* is equal to the number of generations per transfer inferred from the previous step.

#### Whole-genome sequencing and sequence analysis

We performed DNA extractions from evolved MA cell lines using the DNeasy UltraClean Microbial Kit (Qiagen) according to the manufacturer’s instructions, with the additional step of adding 50 μl of 50 mg ml^−1^ lysozyme to improve cell lysis. Genomic DNA was sequenced using Illumina MiSeq sequencing to a depth of at least 35× coverage. Library preparation and DNA sequencing were conducted by the Indiana University Bloomington Center for Genomics and Bioinformatics. Whole-genome sequencing reads were quality controlled using cutadapt^48^ to trim low-quality base pairs and remove residual adapter sequences. We used breseq with the default parameters^49,50^ to call mutations using the trimmed reads. We only considered fixed mutations for the MA cell lines. We checked for mutations that had arisen in experimental ancestor strains before evolution. Ancestral mutations were removed from the analysis of all evolved MA lines derived from that strain using gdtools^49,50^. We used the sequencing data to check for contamination or cross-contamination in the evolved cell lines.

#### Statistical analyses

To compare the mutation rate and spectrum between strains, we used two-sample *t*-tests for numerical response variables and two-sample *χ*^2^ tests with continuity correction for comparing proportions. For comparing proportions to theoretical expectations within a strain, we used one-sample *χ*^2^ tests with continuity correction.

### Adaptive evolution

#### Overview

In contrast to the mutation accumulation experiments, we conducted experiments that allowed bacteria to achieve large population sizes to increase the efficacy of natural selection. This involved serial passaging of cells in liquid cultures with limited bottlenecking at each transfer. For example, in our experiment, the minimum population size was 2× 10^7^–4 × 10^7^ for both JCVI-syn1.0 and JCVI-syn3B. We passaged replicate 3 ml liquid cultures of each strain (*n* = 4 per strain) in 13 mm glass test tubes by 1% (v/v) serial transfer each day for 300 days in a dark, static incubator held at 37 °C. We calculated the number of generations per day as the log_2_ of the dilution factor, that is, log_2_[101], the number of binary fissions needed to regenerate the original population size after the 1% (v/v) transfer^51^. Thus, we estimate that the *M*. *mycoides* strains were maintained for 1,997 generations, which, based on other experiments, is long enough for the majority of adaptation to occur^51,52^.

#### Measurements of fitness

First, we measured fitness as *µ*_max_ by conducting growth curves on cells that were isolated at different timepoints during the adaptive evolution experiment (Supplementary Fig. 5). Cryopreserved cells were thawed on ice before preculturing at 37 °C for 24–72 h in 3 ml of SP4 medium in a 13 mm test tube. Before initiating the experiment, we adjusted the start times of precultures to help ensure that cultures from different evolution timepoints were at the same stage of growth. Approximately 6 × 10^5^ cells from turbid precultures were then inoculated into replicate wells of a 96-well plate containing 200 µl of SP4 medium. Separately, each population was incubated in a 96-well plate for 24 h in a BioTek Synergy H1 microplate reader that recorded the absorbance every 15 min at 415 nm. This wavelength is close to a spectral peak for phenol red, a pH indicator that is a component of SP4 medium (Supplementary Table 3). Previous studies have shown that phenol red can be used as proxy for metabolism and growth^53^ because bacteria like *M. mycoides* produce organic acids as a byproduct of carbohydrate metabolism^4^ (Supplementary Fig. 5). With the resulting data, we used maximum likelihood to estimate growth-curve parameters using a modified Gompertz equation^54^:

$$Y={b}_{0}+A\times \exp \left\{-\exp \left[\frac{{\mu }_{\max }\times {\rm{e}}}{A}\left(L-t\right)+1\right]\right\}$$

where *L* is the lag time (h), *A* is the carrying capacity or yield (optical density at 415 nm), *µ*_max_ is the maximum growth rate (day^−1^) and *b*_0_ is the intercept (Supplementary Fig. 6 and Supplementary Table 5).

Second, we measured relative fitness by competing ancestral and evolved strains against a *M. mycoides* JCVI-syn1.0 reference strain labelled with mCherry (syn1.0::mCh)^26^. Cryopreserved cells were used to make precultures in a similar manner to those in the growth curve experiment. Each strain was grown in liquid medium to log phase, and then the labelled and unlabelled strains were simultaneously diluted into a mixed culture in fresh medium. We immediately sampled the axenic cultures or the mixed culture (*t*_0_), fixed the cells with 20 μl of cold 25% glutaraldehyde, incubated them at 4 °C for 20 min and then stained the samples with 2× SYBR Green. After 24 h of growth (*t*_*f*_), the mixed culture was sampled and processed again in an identical manner. For samples in the adaptive evolution experiment, we quantified the abundance of each strain using a an LSR II flow cytometer (BD Biosciences) at Indiana University’s Flow Cytometry Core Facility. For measuring the relative fitness of engineered *ftsZ* mutants, we used the NovoCyte flow cytometer (ACEA Biosciences). While measurements were being made, we vortexed the samples every minute to prevent multiple cells from clumping together and being scored as single events. The purity was assessed during every run using negative controls and axenic controls. We detected 1,800–2,700 events per second and abundances on the order of 1 × 10^8^ cells per ml. With the resulting data, we differentiated cells on the basis of the expression of mCherry. Using NovoExpress, FACSDiva and FCS Express software, we established gates on pure cultures of the non-mCherry-expressing experimental strains and the syn1.0::mCh reference strain (Supplementary Figs. 7 and 8). For the experimental strains, boundaries were established by gating axenic mCherry-negative cells that were positive for only SYBR Green fluorescence. For the reference strain, boundaries were established by gating axenic syn1.0::mCh cells that were positive for SYBR Green and mCherry (Supplementary Fig. 9). In the competition assays used to quantify relative fitness, we applied the axenically established gates to samples that contained a mixture of the reference strain and experimental strain. We obtained the proportion of false-negative mCherry cells by applying the mCherry-negative gate to axenic mCherry-expressing cells; this proportion was then used as a correction factor in mixed populations. Last, we calculated relative fitness as the change in the relative abundance of the strain of interest during the 24 h period of competitive growth versus syn1.0::mCh. Specifically, the relative fitness versus the mCherry reference strain *W*_*C*_ is

$${W}_{C}=\frac{{\rm{ln}}\left(\frac{{N}_{{\rm{f}}}}{{N}_{0}}\right)}{{\rm{ln}}\left(\frac{{N}_{{\rm{Cf}}}}{{N}_{{\rm{C}}0}}\right)}$$

where *N*_0_ represents the initial abundance of the experimental strain, *N*_f_ the abundance of the experimental strain after 24 h, and *N*_C0_ and *N*_Cf_ are initial and final abundances of the reference strain (syn1.0::mCh), respectively^26^. We normalized fitness values to be relative to the original *M. mycoides* JCVI-syn1.0 ancestor strain. In other words, we represent the fitness (*W*) as $\frac{{W}_{C}}{{W}_{{\rm{J}}{\rm{C}}{\rm{V}}{\rm{I}}-{\rm{s}}{\rm{y}}{\rm{n}}1.0}}$, where *W*_JCVI -syn1.0_ is the value of *W*_*C*_ for *M. mycoides* JCVI-syn1.0.

#### Whole-genome sequencing and sequence analysis

DNA extraction, sequencing and bioinformatics were performed according to the same methods as for the mutation accumulation experiment with a few exceptions. Specifically, each replicate population was sequenced to a depth of at least 100× coverage, and polymorphic mutations were included in our analyses. As an indicator of selective pressure, we used the Jukes–Cantor method^55^ to compute the per-site *d*_N_/*d*_S_ value on the basis of the number of nonsynonymous and synonymous SNMs within each of the evolved replicate populations normalized by the total nonsynonymous and synonymous target sizes. We counted the number of synonymous and nonsynonymous AT to CG, AT to GC, AT to TA, CG to GC, CG to TA and CG to AT sites using the gdtools module of breseq, which is a computational pipeline that identifies mutations from short-read DNA resequencing studies^50^. We next combined that information with the empirical mutation spectrum from the MA experiment to account for the differing probabilities of each of the six SNM types, and thereby calculate the total expected number of SNMs at nonsynonymous and synonymous sites^56^. The observed numbers of synonymous and nonsynonymous substitutions were obtained directly from breseq outputs. Synonymous and nonsynonymous polymorphisms were included in the observed count with probability equal to their allele frequency in mapped reads. We added a pseudocount of 1 synonymous substitution for all calculations^57^ because two of the populations had 0 synonymous substitutions.

To identify mutations possibly contributing to adaptation, we looked for genes that had mutations across two or more replicate populations for each genotype. Mutations in the same gene, arising and increasing in frequency in independent lineages, suggests that that mutation’s rise could be driven by positive selection^58^. To test this hypothesis, we statistically assessed whether multiply-mutated genes (that is, genes mutated in >1 replicate evolved population) had acquired more mutations than would be expected by chance under the assumption that the mutations were neutral^58^. To do this, we recorded all of the polymorphic and fixed mutations that were called within genes. Synonymous mutations were excluded. We then used Python^59^ to simulate the placement of these mutations at random across all genes. The probability of any given gene receiving any given mutation was relativized to the gene’s length and GC content using the known mutation rates of G:C nucleotides and A:T nucleotides from the mutation-accumulation experiment. We repeated this random placement of mutations 100,000 times. In each simulation, we counted the number of mutations received by each gene, with each fixed mutation increasing the count by 1 and each polymorphism increasing the count by an amount equal to its allele frequency. For each multiply-mutated gene from the real adaptation experiment, we calculated the proportion of the 100,000 simulations in which the gene received at least as many mutations as were truly observed and called this proportion the *P* value. We then used the Benjamini–Hochberg method^60,61^ to generate corrected *P* values (*P*_adj_) to account for multiple tests with the false-discovery rate set to be *α* = 0.05 (Extended Data Table 2). As a negative control, we repeated the simulations using only synonymous mutations. This process returned two false-positive significant genes, which was small compared with the 52 significant signatures detected among nonsynonymous mutations, although we also acknowledge that synonymous gene analysis had less power due to the smaller number of synonymous mutations.

#### Generation of *ftsZ* E315* mutant cells

This process required mutating the bacterial genomes while they were yeast centromeric plasmids (YCPs) followed by genome transplantation of the mutated genomes. The YCPs were mutated using rounds of CRISPR–Cas9 and yeast homologous recombination that is a modification of a method used previously to mutate *M. mycoides* strains^62^.

In the first CRISPR–Cas9 step, the molecule to be mutated was cleaved and the donor DNA comprising sequences from the two flanking genes was recombined with the cut JCVI-syn1.0 or JCVI-syn3B YCP, removing parts of genes of the flanking genes and all of the target gene. The donor DNA had 40 bp overlaps to both genes flanking the target gene and had a 22 bp *Mycoplasma gallisepticum* 161 CRISPR–Cas9 target sequence with a protospacer adjacent motif (PAM) (5′-GTATAAATACATCCAGGAGTGG-3′) that had no homology elsewhere in JCVI-syn1.0 or JCVI-syn3B. The *M. gallisepticum* sequence put a new PAM in the genome that was used in the second round of CRISPR–Cas9.

The second round of CRISPR–CAS9 cut the JCVI-syn1.0 or JCVI-syn3B YCP at the new *M. gallisepticum* PAM. The cut YCP was then recircularized using a donor DNA containing the desired point mutation. The mutagenized regions of the YCPs were PCR amplified and the mutation was confirmed by Sanger sequencing. Correctly mutagenized JCVI-syn1.0 or JCVI-syn3B YCPs were then transplanted into *Mycoplasma capricolum* recipient cells as reported previously^3,59,60,63,64^. The mutagenized regions of the transplants were PCR-amplified and sequenced to confirm the presence of the desired mutations.

#### Microscopy and image analysis

Scanning electron microscopy (SEM) was used to compare changes in the cell size of evolved populations. All of the populations were grown in the same batch of medium and under identical conditions in a single incubator. The start times of cultures were adjusted so that they reached stationary phase at the same time. We centrifuged stationary-phase cultures and resuspended the pellet in 1 ml of phosphate-buffered saline (pH 7.4). The resuspended cells were fixed by adding 20 μl of cold 25% glutaraldehyde and incubating at 4 °C for 20 min. For microscopy observation, fixed cells were concentrated 4× by centrifugation and resuspension. The centrifugation steps were performed at 25 °C for 4 min at 2,000*g*. SEM was performed at the Indiana University Bloomington Electron Microscopy Center. Fixed cells in PBS were pelleted and resuspended in 100 mM sodium cacodylate buffer (pH 7.2) with 2 mM calcium chloride and 2% sucrose. We coated 12-mm-diameter glass coverslips with 0.1% poly-l-lysine for 5 min, after which coverslips were washed with a few drops of double distilled water. Resuspended cells were added to the coverslip surface and allowed to adhere. After 5 min, the coverslips were washed twice with 100 mM sodium cacodylate buffer (pH 7.2) with 2  mM calcium chloride and 2% sucrose. Next, 300 µl of 2% osmium tetroxide in 100 mM sodium cacodylate buffer (pH 7.2) with 2% sucrose was added to the surface of the coverslips while on ice. After 30 min, the coverslips were washed with double-distilled water. The coverslips were placed into a CPD coverslip holder (Electron Microscopy Sciences, 70193-01). The samples were dehydrated in a graded ethanol series (30%, 50%, 70%, 90%, 95%) while on ice. At room temperature, the coverslips were rinsed three times with 100% ethanol. Each dehydration step lasted for 2 min. Critical-point drying was performed using the Tousimis Samdri 790 critical-point dryer. The dried coverslips were placed on aluminium SEM stubs and sputter-coated using the Safematic CCU-010 with SP-010 Sputter Head with 45 nm of gold/palladium (80%/20%), which is accurate in the Angstrom range. All of the samples were coated simultaneously to minimize variance among samples. We viewed the samples using the FEI Teneo scanning electron microscope at 2.0 kV, 25 pA probe current and 3.0 mm working distance. The T2 detector was used. We calibrated the measurements using line grating replicas (2,160 lines per mm) with 0.261 μm latex spheres (Electron Microscopy Sciences). We analysed the SEM image data using ImageJ^65^. We used the straight and measure features combined with image scale metadata to measure the vertical diameters of imaged cells that met the following criteria: cells must be round; cells must not have apparent holes or punctures; cells must be completely within the field of view; cells must have an unambiguous perimeter; there must be no suggestion that a cell is currently or has recently undergone binary fission; cells must be ≥0.1 μm across. Each image was processed counterclockwise starting from east. The samples were processed in a randomized order.

#### Statistical analyses

For the growth-curve experiments, we used a generalized linear mixed model to test for the fixed effects of time (generation) and cell type (minimal versus non-minimal) on growth curve parameters (*µ*_max_, lag time, yield) while fitting random intercepts for the replicate evolved populations (Supplementary Table 3). We used variance partition coefficients to estimate the contribution of the replicate populations (random effect) to the total variation explained in the models (Extended Data Fig. 1, Extended Data Table 1, Supplementary Figs. 1 and 2 and Supplementary Table 5). For the adaptative evolution experiment (Figs. 2 and 4), we tested hypotheses using a general linear model (GLM) after subtracting observations of each replicate-evolved population (generation 2,000) from its corresponding ancestor (generation 0). With the intercept term excluded, the GLM tests whether the evolutionary trajectory for each group is different from zero. With the intercept term included, the GLM tests whether the evolutionary trajectories are different among groups. We also used two-way ANOVA with Tukey’s honest significant difference test to test hypotheses about the effects of cell type (minimal versus non-minimal) and *ftsZ* E315* (wild type versus mutant) on relative fitness and cell size. When necessary, data were log_10_-transformed to meet statistical assumptions.

We compared the composition of genes acquiring mutations among the evolved replicate populations by first constructing a gene-by-population matrix. Here, each row represented an evolved population and each column represented a gene that had acquired at least one mutation among all of the populations. Each cell of the matrix was filled with the sum value of mutations occurring in that gene in that population, where fixed mutations were valued at 1 and polymorphisms were valued equal to the allele frequency. Only essential genes, shared between JCVI-syn1.0 and JCVI-syn3B, were considered. We used PERMANOVA on the Bray–Curtis distances generated from the gene-by-population matrix to test for the significance of cell type (minimal versus non-minimal) on the composition of mutations using the adonis function in the R package vegan^66^. For visualization, the Bray–Curtis distances were decomposed into two dimensions using principal coordinate analysis using the cmdscale function.

### Reporting summary

Further information on research design is available in the Nature Portfolio Reporting Summary linked to this article.

## Online content

Any methods, additional references, Nature Portfolio reporting summaries, source data, extended data, supplementary information, acknowledgements, peer review information; details of author contributions and competing interests; and statements of data and code availability are available at 10.1038/s41586-023-06288-x.

### Supplementary information

Supplementary Information Supplementary Figs. 1–9, Supplementary Tables 1–5 and two citations supporting the experimental and statistical procedures described in the main manuscript.

Reporting Summary

Supplementary Data 1 Delta fitness trajectory data for relative fitness in Supplementary Fig. 1.

Supplementary Data 2 *d*_N_/*d*_S_ data for non-minimal cell in Supplementary Fig. 2.

Supplementary Data 3 Data for the percentage change in cell size in Supplementary Fig. 3.

Supplementary Data 4 Delta size trajectory data for cells in Supplementary Fig. 4.

Supplementary Data 5 Growth curve data for Supplementary Fig. 5.

Supplementary Data 6 Growth curve data for Supplementary Fig. 6.

Supplementary Data 7 Flow cytometry data for Supplementary Fig. 7.

Supplementary Data 8 Flow cytometry data for Supplementary Fig. 8.

Supplementary Data 9 Flow cytometry data for Supplementary Fig. 9.

### Source data

Source Data Fig. 1

Source Data Fig. 2

Source Data Fig. 3

Source Data Fig. 4

Source Data Extended Data Fig. 1

Source Data Extended Data Fig. 2

Source Data Extended Data Fig. 3

Source Data Extended Data Fig. 4

## Figures and Tables

**Extended Data Table 1:**
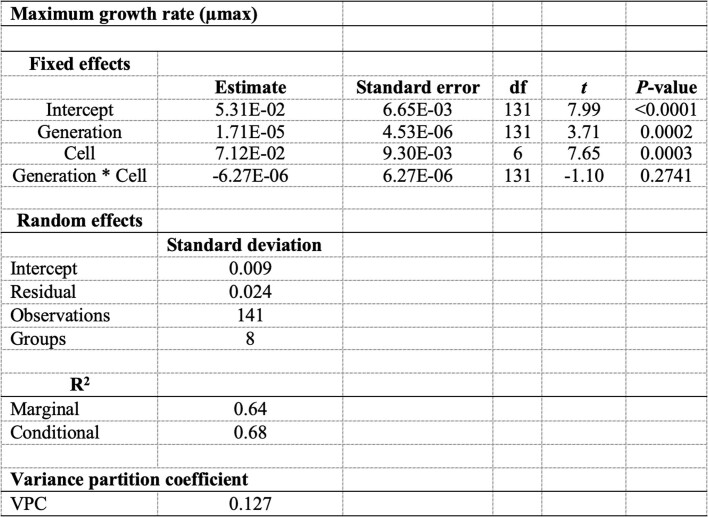
Parameters and summary statistics associated with fitness

We used generalized linear mixed models (GLMM) to explain variation in maximum growth rate (*µ*max), which was estimated from growth curves for the non-minimal and minimal cells. The cell type (“Cell”) and time (“Generation”) were treated as fixed effects. We included random intercepts for the replicate populations.

**Extended Data Table 2:**
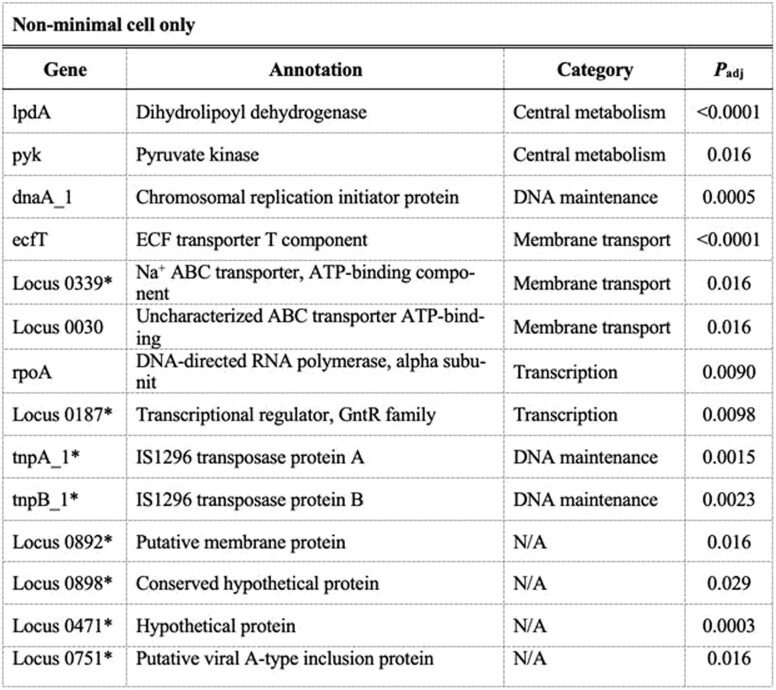
Mutations only in non-minimal cell that that are putatively under positive selection

Simulations were performed to find genes acquiring more mutations than expected to occur by chance during 2000 generations of experimental evolution. Such mutations are indicative of positive selection. *P*_*adj*_ corresponds to significance following Benjamini-Hochberg correction to account for multiple comparisons. Genes are assigned to categories based on the secondary functional classifications^4^. Note that “Central metabolism” corresponds to “Central carbon metabolism” in the original source^4^. * = nonessential genes that are absent from the minimal cell, N/A = uncharacterized genes that did not fall into defined category.

**Extended Data Table 3:**
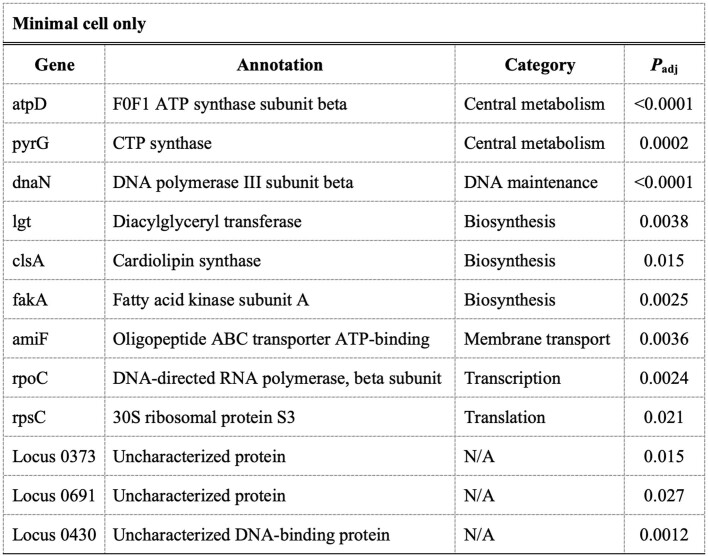
Mutations only in minimal cell that are putatively under positive selection

Simulations were performed to find genes acquiring more mutations than expected to occur by chance during 2000 generations of experimental evolution. Such mutations are indicative of positive selection. *P*_*adj*_ corresponds to significance following Benjamini-Hochberg correction to account for multiple comparisons. Genes are assigned to categories based on the secondary functional classifications^4^. Note that “Central metabolism” corresponds to “Central carbon metabolism” in the original source^4^.

**Extended Data Table 4:**
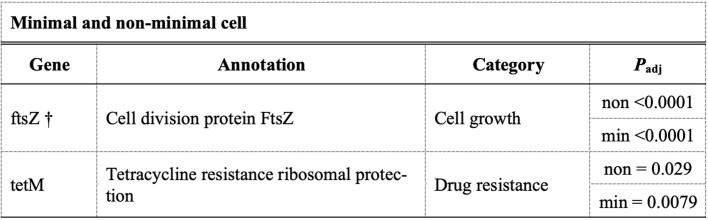
Mutations found in both non-minimal and minimal cell that are putatively under positive selection

Simulations were performed to find genes acquiring more mutations than expected to occur by chance during 2000 generations of experimental evolution. Such mutations are indicative of positive selection. *P*_*adj*_ corresponds to significance following Benjamini-Hochberg correction to account for multiple comparisons. Genes are assigned to categories based on the secondary functional classifications^4^. In the *P*_adj_ column, “non” refers to non-minimal cell and “min” refers to the minimal cell. † = nonessential genes that were retained in *M. mycoides* JCVI-syn3B to facilitate cultivation and robust growth.

**Extended Data Table 5:**
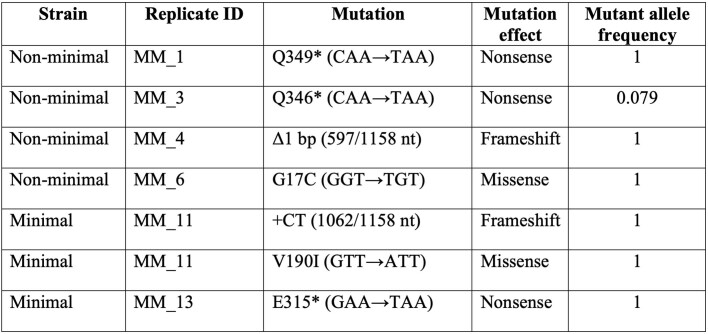
*ftsZ* mutations

Mutations observed in *ftsZ* during adaptive evolution across all replicate populations.

**Extended Data Table 6:**
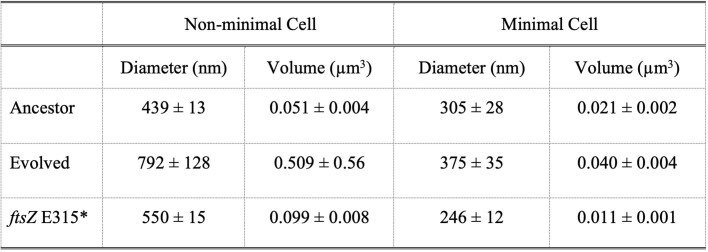
Evolution of cell size

Diameter and volume measurements of *M. mycoides* ancestor, evolved, and *ftsZ* E315* cells observed *via* scanning electron microscopy. Values represent mean ± SEM.

## Data Availability

Data are available at GitHub (https://github.com/LennonLab/MinimalCell), Zenodo (10.5281/zenodo.7953578), Figshare (10.6084/m9.figshare.23119985) and the NCBI Sequence Read Archive (PRJNA743406). Source data are provided with this paper.

## Code availability

Computing code for reproduction is available at GitHub (https://github.com/LennonLab/MinimalCell) and Zenodo (10.5281/zenodo.7953578).
