# Publisher Correction: Evolution of a minimal cell

**DOI:** 10.1038/s41586-023-06454-1

**Published:** 2023-07-26

**Authors:** R. Z. Moger-Reischer, J. I. Glass, K. S. Wise, L. Sun, D. M. C. Bittencourt, B. K. Lehmkuhl, D. R. Schoolmaster, M. Lynch, J. T. Lennon

**Affiliations:** 1 Department of Biology, Indiana University, Bloomington, IN USA; 2 J. Craig Venter Institute, La Jolla, CA USA; 3 Novartis Gene Therapy, San Diego, CA USA; 4 Embrapa Genetic Resources and Biotechnology, National Institute of Science and Technology in Synthetic Biology, Brasília, Brazil; 5 US Geological Survey, Wetland and Aquatic Research Center, Lafayette, LA USA; 6 Arizona State University, Tempe, AZ USA

**Keywords:** Experimental evolution, Bacterial genes, Genome evolution, Synthetic organisms, Molecular evolution

Correction to: *Nature* 10.1038/s41586-023-06288-x Published online 5 July 2023

In the version of this article initially published, B. K. Lehmkuhl (Department of Biology, Indiana University, Bloomington, IN, USA) was shown with an incorrect affiliation, and Extended Data Table 2 was a duplicate of Extended Data Table 3. The errors have been corrected in the HTML and PDF versions of the article.

