# Supplementary material for: Evolution of a minimal cell
Source: Nature. 2023 Jul 5;620(7972):122–7. doi: 10.1038/s41586-023-06288-x (PMC10396959; doi:10.1038/s41586-023-06288-x)
Supplement: Supplementary file 1 — Supplementary Figs. 1–9, Supplementary Tables 1–5 and two citations supporting the experimental and statistical procedures described in the main manuscript. [file 41586_2023_6288_MOESM1_ESM.docx]

**Supplementary Information:**

**Evolution of a minimal cell**

Moger-Reischer RZ^1^, Glass JI^2^, Wise KS^2^, Sun L^2,3^, Bittencourt DMC^2,4^, Lehmkuhl BK, Schoolmaster Jr DR^5^, Lynch M^6^, Lennon JT^1*^

^1^ Department of Biology, Indiana University, Bloomington, IN 47405, USA

^2^ J. Craig Venter Institute, La Jolla, CA 92037, USA

^3^ Novartis Gene Therapy, San Diego, CA 92121 USA

^4^ EMBRAPA Genetic Resources and Biotechnology, Brasília, 70770-917, Brazil

^5^ U.S. Geological Survey, Wetland and Aquatic Research Center, Lafayette, LA 70503 USA

^6^ Arizona State University, Tempe, AZ 85287, USA

* Corresponding author: [lennonj@indiana.edu](mailto:lennonj@indiana.edu)

**
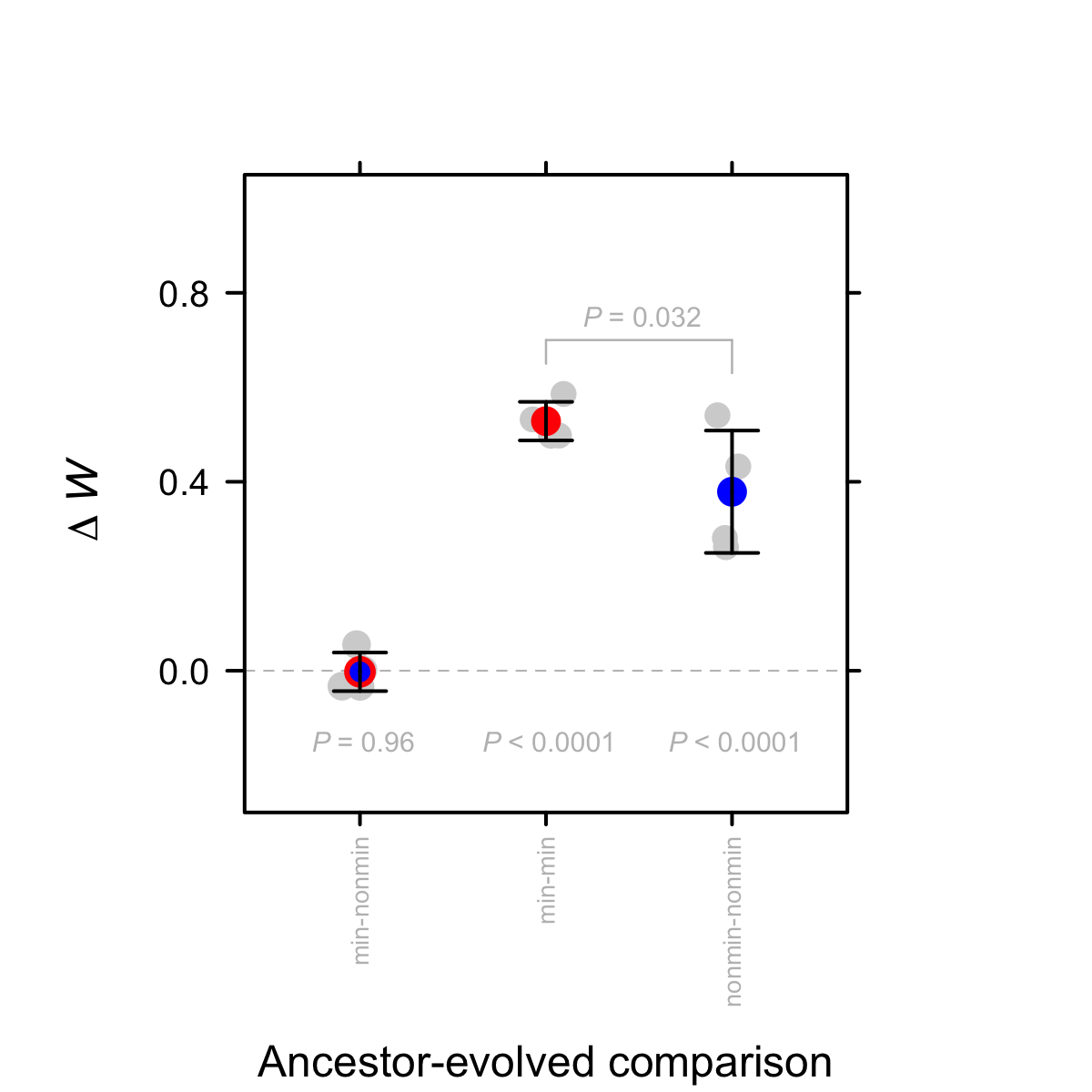
**

**Fig. S1. Fitness trajectories.** Changes in relative fitness (Δ*W*) estimated by subtracting the relative fitness values on replicate evolved populations (n = 4) from the relative fitness value of the ancestor (n =1). The figure is based on data in Fig. 2 in the main text. There were three ancestor-evolved comparisons that were relevant to the study (x-axis). From left to right, we first compared the relative fitness of the evolved minimal cell to the relative fitness of the ancestral non-minimal cell to quantify the degree to which the minimal cell recovered from genome streamlining after 2,000 generation (`min-nonmin`). Second, we compared the relative fitness of the evolved minimal cell to the relative fitness of the ancestral minimal cell (`min-min`). Third, we compared the relative fitness of the evolved nonminimal cell to the relative fitness of ancestral nonminimal cell (`nonmin-nonmin`). We then used a generalized model (GLM) to test for differences. With the intercept term excluded, the GLM tested whether Δ*W* for each group is different from zero (*P*-values below the x-axis). With the intercept term included, the GLM tested whether Δ*W* is the same for the minimal cell and non-minimal cell (*P*-value above line segment connecting `min-min` vs. `nonmin-nonmin` comparison). Grey symbols represent raw Δ*W* values; colored symbols represent the means of the Δ*W* values; error bars represent ± 95% confidence intervals.


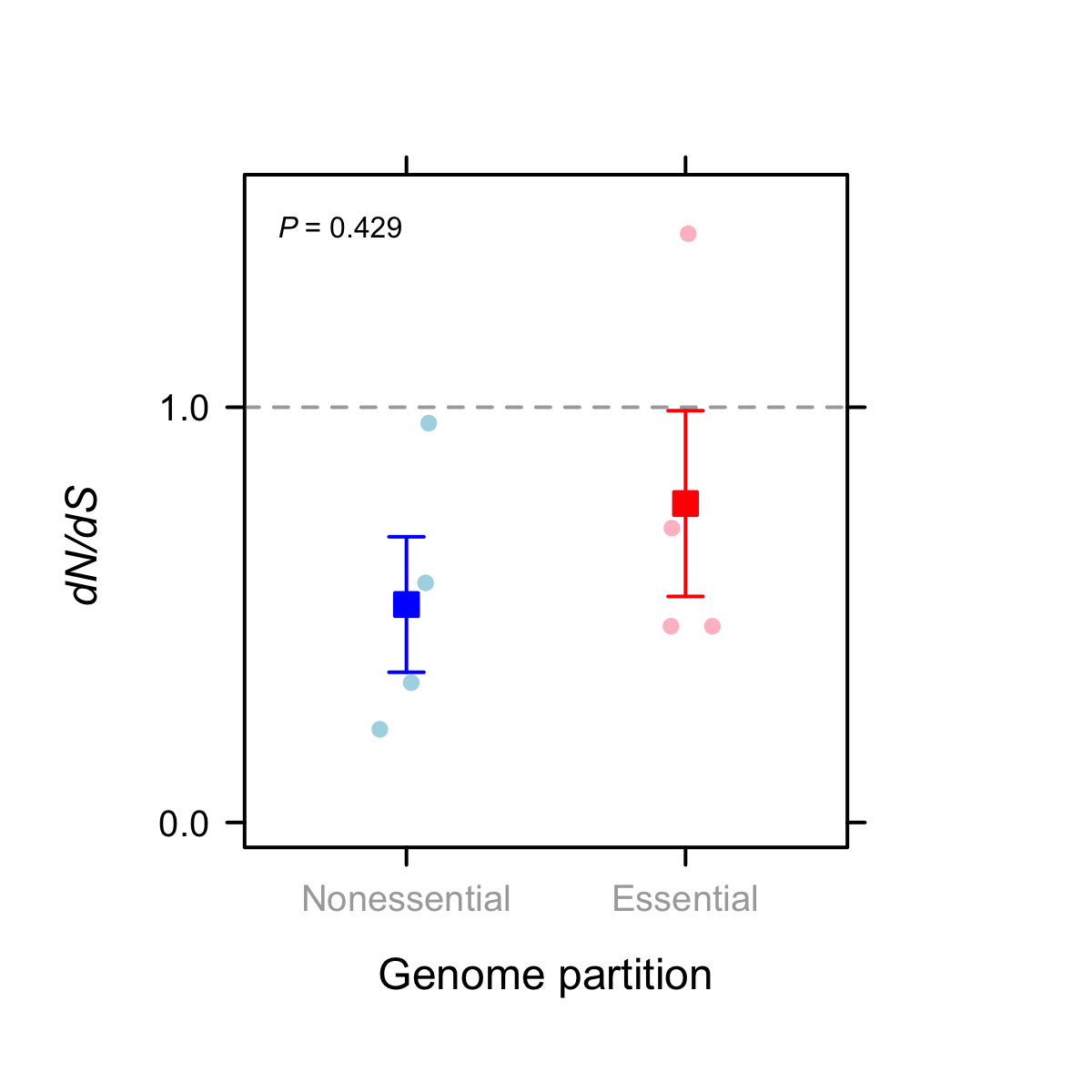


**Fig S2***.* ***d_N_/d_S_* of non-minimal cell.** Effect of genome streamlining on the ratio of nonsynonymous to synonymous substitutions between the nonessential and essential partitions of the non-minimal cell’s genome. The normalized ratio of nonsynonymous to synonymous mutations (*d_N_*/*d_S_*) can indicate the relative action of positive or negative selection. Values of *d_N_*/*d_S_* > 1 indicate positive selection, while *d_N_*/*d_S_* < 1 can indicate the dominance of negative selection and constraint on adaptation. With the experimental design (n = 8), there was no significant effect of genome minimization on *d_N_*/*d_S_* between the two types of genes. Dark-colored symbols represent the mean ± SEM. Light-colored symbols represent values for each replicate population. *P*-value corresponds to output from a two-sample *t*-test.


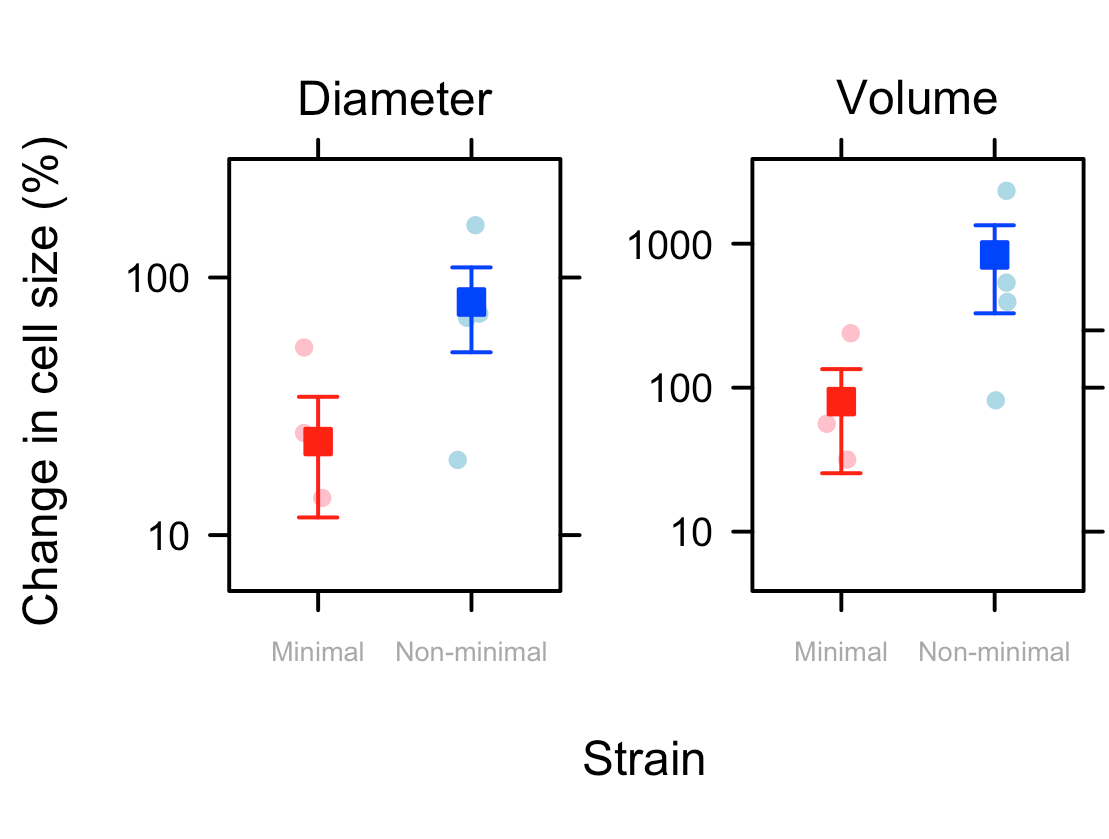


**S3**. **Minimization and cell size.** Effect of genome minimization on percent change in cell size for the minimal cell (red) and non-minimal cell (blue) over 2000 generations of experimental evolution. Dark-colored symbols represent mean ± SEM for replicate populations (n = 4). Light-colored symbols represent values for each replicate population.

**
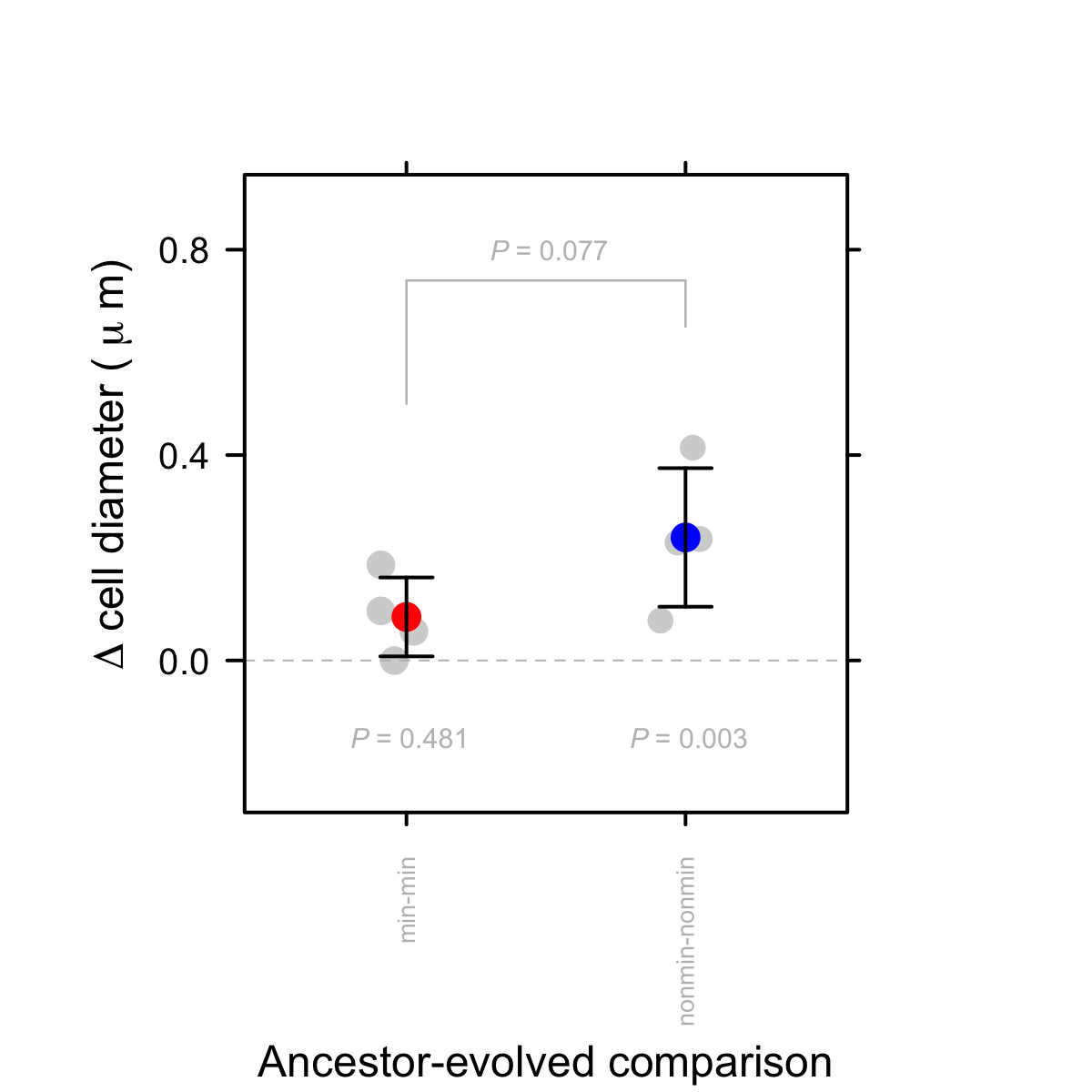
**

**Fig. S4. Cell size trajectories.** Changes in cell size (Δ cell diameter) estimated by subtracting the mean size of cells from replicate evolved populations (n = 4) from the mean size of cells of its ancestor (n =1). The figure is based on data in Fig. 4 of the main manuscript. There were two ancestor-evolved comparisons that were relevant to the study (x-axis). First, we compared the size of the evolved minimal cell to the size of ancestral minimal cell (`min-min`). Second, we compared the size of the evolved nonminimal cell to the size of ancestral nonminimal cell (`nonmin-nonmin`). We then used a generalized model (GLM) to test for differences. With the intercept term excluded, the GLM tested whether Δ cell diameter for each group is different from zero (*P*-values below the x-axis). With the intercept term included, the GLM tested whether Δ cell diameter is the same for the minimal cell and non-minimal cell (*P*-value above line segment connecting `min-min` vs. `nonmin-nonmin` comparison). Grey symbols represent raw Δ cell diameter values; colored symbols represent the means of the Δ cell diameter values; error bars represent ± 95% confidence intervals.


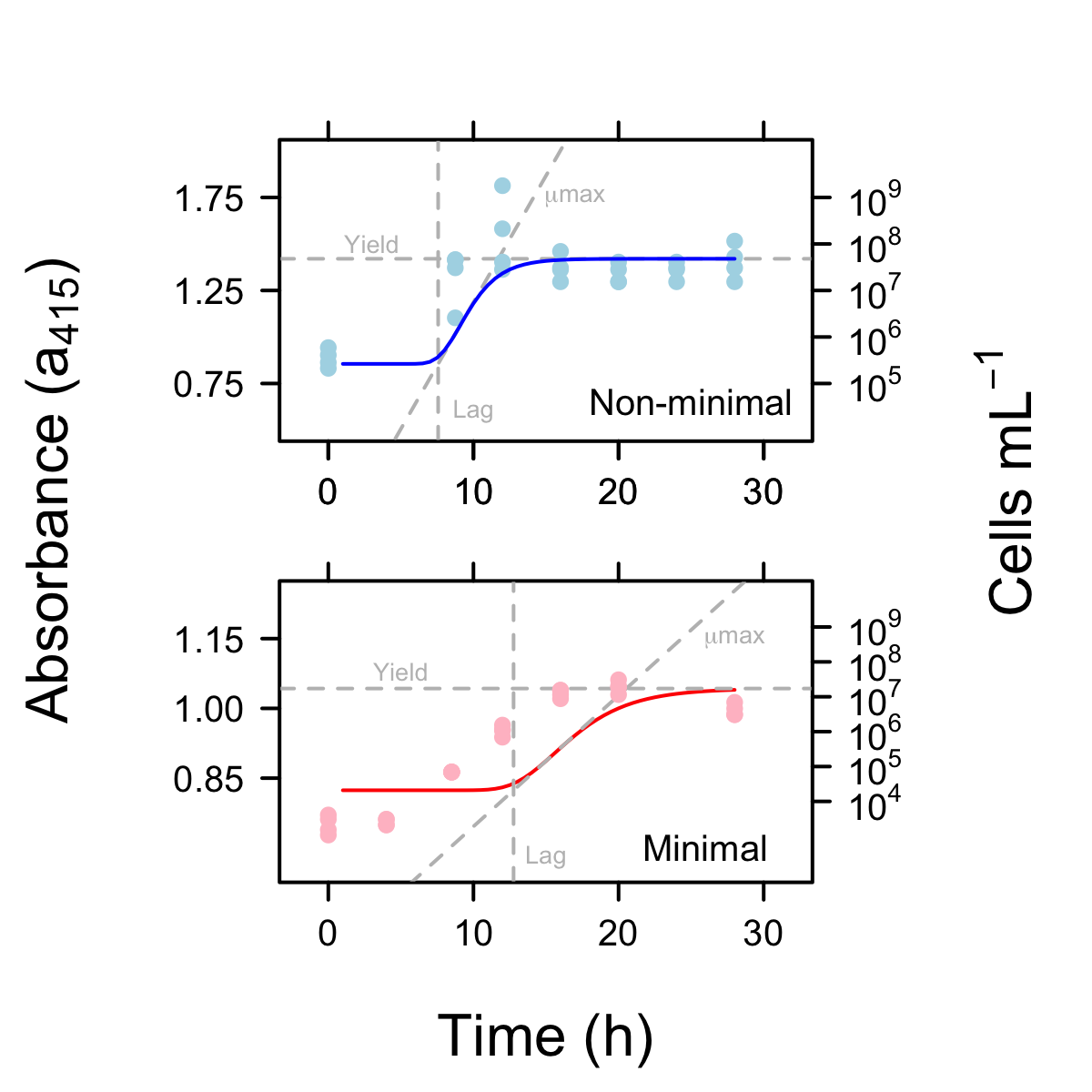


**Fig. S5**. ***Mycoplasma* growth curves.** We conducted growth curve assays on cultures of the minimal cell and non-minimal cell taken from replicate populations in the 2000-generation evolution experiment. Presented here are examples of growth curves for the non-evolved ancestor of the non-minimal (top) and the minimal cell (bottom). Dilute suspensions of pre-cultured cells were inoculated into 96-well plates with SP4 medium containing phenol red, a pH indicator. Color change in the medium due to growth and metabolism associated with organic acid production by *Mycoplasma* when fermenting carbohydrates can be detected with a spectrophotometric 96-well plate reader as absorbance at 415 nm (see Methods). We then used a modified Gompertz model to fit the high-resolution absorbance data. The solid blue and red lines represent the average predicted growth curves for 47 and 34 replicates, respectively, along with the corresponding averages of the Gompertz-derived growth parameters: yield (optical density at 415 nm), µmax (d^-1^), and lag time (h). Additional information, including model fits, parameters, summary statistics, and residual plots can be found in the online Figshare repository. These reports reveal that the Gompertz model did a good job of fitting absorbance data (mean coefficient of variation = 1.9% and mean of root mean square error = 0.025). Furthermore, variation among technical replicates (*n* = 3) for a given culture was low for µmax = 0.10 %, yield = 0.56%, and lag time = 8.65%. Previous findings have demonstrated that phenol red is a reliable measure of metabolism and growth for *Mycoplasma* strains^1^. Nevertheless, we validated the approach by measuring population sizes of *Mycoplasma* at the same time as absorbance reading were being taken with the plate reader. We used most probable number (MPN) method, which involved 10-fold serial dilution-to-extinctions of replicate samples at each time point based on binary changes in color associated with phenol red indicator^2^. While not as reliable an estimate, the population size estimates from the MPN approach generally agree with higher resolution plate-reader data. The MPN data, which are represented by the light blue (non-minimal) and pink (minimal) circles, are not being fit to the Gompertz model in this figure; they are included only as reference points.


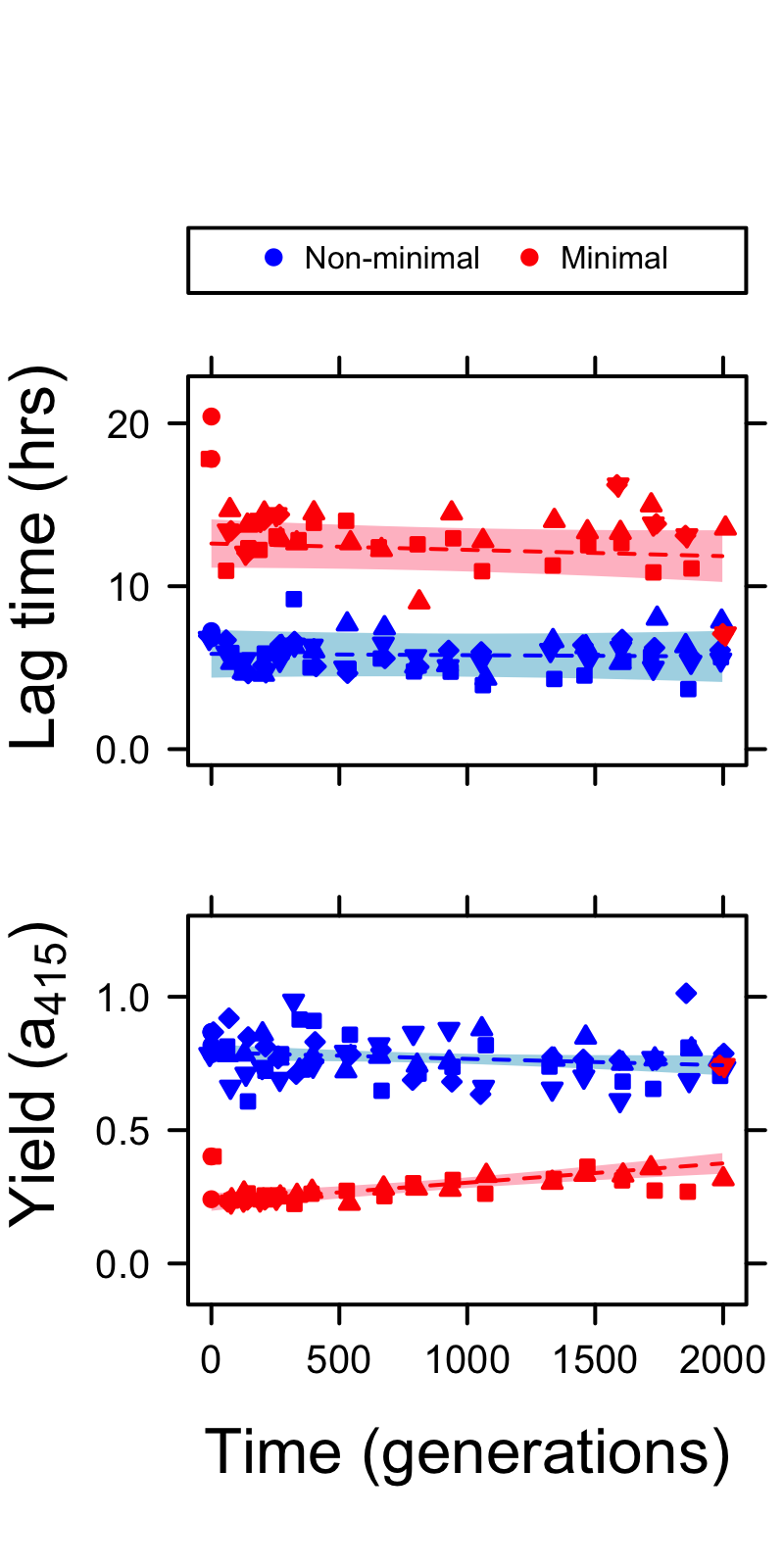


**Fig. S6**. **Trajectories of growth curve parameters.** Lag time and yield for the minimal cell and non-minimal cell over 2000 generations of experimental evolution. Data were generated from growth curve assays that were fit using a modified Gompertz equation (see Fig. S5). As in Extended Data Fig. 1, we then fit a generalized linear mixed model (GLMM) where time (generation) and cell type (minimal cell vs. non-minimal cell) were treated as fixed effects and replicate evolved populations (n = 8) was treated as a random effect. In the figure, dark-colored circles represent data from the ancestral populations, while triangles (up- and down-pointing), diamonds, and squares represent data from the replicate evolved populations. Dashed lines and light-colored regions represent predicted values and 95% confidence intervals, respectively, for the fixed effects (generation and cell type). A summary of parameters and summary statistics for the GLMM can be found in Table S6. An online Figshare repository contains model fits, parameters, summary statistics, and residual plots. In addition to testing trends over time, we also explored correlations among growth curve parameters as this could provide additional insight into the fitness gains of the minimal and non-minimal cell during the evolution experiment. For the minimal cell, we found that yield increased with µmax (*r* = 0.77, *t*_67_ = -7.66, *P* = 9.8 x 10^-11^) and that lag time decreased with increasing µmax (*r* = -0.47, *t*_67_ = -4.34, *P* = 5.1 x 10^-5^. Fitness alignment for these growth parameters was less strong for the non-minimal cell. For example, yield was not correlated with µmax (*r* = -0.06, *t*_70_ = -0.51, *P* = 0.609) and lag time actually increased with µmax (*r* = 0.39, *t*_70_ = 3.58, *P* = 0.0006). These findings are consistent with the interpretation that streamlining does not constrain fitness evolution of populations over time, and may actually create opportunities for rapid adaptation. It is important to note that there are important distinctions when measuring fitness from growth curves (e.g., µmax, lag time, and yield) and measuring fitness from competition assays. Relative fitness is the net result of competition among strains. The outcome of competition is determined by a suite of traits. For example, in addition to µmax, traits like per capital mortality and resource use efficiency likely contribute to relative fitness. The complete recovery of relative fitness without an equal and proportional change in maximum growth rate suggests there are other parameters associated with competitive ability that should be investigated.


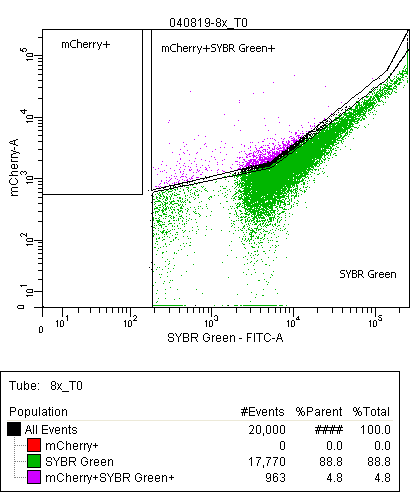


**Fig. S7. Flow cytometry gating.** Example of a flow cytometric gating strategy for axenic non-fluorescent *Mycoplasma mycoides* without mCherry gene (negative). Data in scatter plot are represented on an absolute scale. Table below the graph displays population statistics. “All Events” includes three categories. “mCherry+” represents the expected fluorescence for an mCherry-expressing cell not stained with SYBR Green. “SYBR Green” events represent non-mCherry-expressing, SYBR Green-stained cells. “mCherry+SYBR Green+” represent mCherry-expressing events if there were any such cells in a (mixed) population. “#Events”: Number of events within the parameters of that corresponding gate. “% Parent”: Percentage of the total events that fell within the parameters of that particular gate. “%Total”: Percentage of the total events that fell within the parameters of that particular gate.


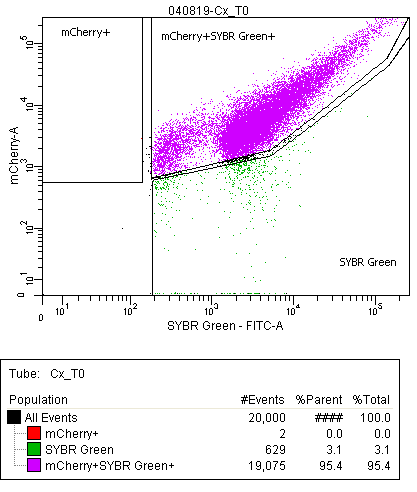


**Fig. S8. Flow cytometry gating.** Example of a flow cytometric gating strategy for axenic fluorescent *Mycoplasma mycoides* with mCherry gene (positive). Data in scatter plot are represented on an absolute scale. Table below the graph displays population statistics. “All Events” includes three categories. “mCherry+” represents events with the expected fluorescence for an mCherry-expressing cell not stained with SYBR Green. “SYBR Green” represents cells stained with SYBR Green that do not express mCherry. In this axenic population, they represent the amount of false-negative misclassifications. “mCherry+SYBR Green+” represents mCherry-expressing cells that express the fluor sufficiently to be correctly characterized as such. “#Events”: Number of events within the parameters of that corresponding gate. “%Parent”: Percentage of the total events that fell within the parameters of that particular gate. “%Total”: Percentage of the total events that fell within the parameters of that particular gate.


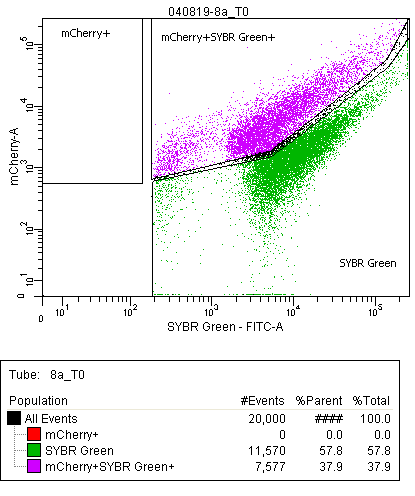


**Fig. S9**. **Flow cytometry gating.** Example of a flow cytometric gating strategy for a mixed population of *Mycoplasma mycoides* comprising cells with (positive) and without (negative) the mCherry gene. We copied the absolute-scale gates from the positive and negative populations. We used the empirical proportion of false negative cells to apply a correction to the inferred number of true negative cells. Inset table represents population statistics. “All Events” includes three categories. “mCherry+” events within this gate would represent the expected fluorescence for an mCherry-expressing cell not stained with SYBR Green. “SYBR Green” represents cells stained with SYBR Green that do not express mCherry. This includes both “true negative” non-fluorescent cells and “false negative” cells that express mCherry but insufficiently in order to be measured as such. “mCherry+SYBR Green+” represent mCherry-expressing cells that express the fluor sufficiently to be correctly characterized as such. “#Events”: Number of events within the parameters of that corresponding gate. “%Parent”: Percentage of the total events that fell within the parameters of that particular gate. “%Total”: Percentage of the total events that fell within the parameters of that particular gate.

**Table S1**. ***d_N_/d_S_* of non-minimal cell.** Values of *d_N_*/*d_S_*, mutation counts, and target sizes for the essential and nonessential partitions of the non-minimal genome. *d_N_*/*d_S_* was calculated after accounting for the empirical mutation spectrum, as described in the Methods. Mutation count was calculated by summing the number of genic mutations in each population that fell within either essential or nonessential genes. Fixed mutations were counted at value = 1, while polymorphisms were counted at value equal to the allele frequency. Partition size was the number of essential or nonessential genes. Relativized mutation count among nonessential genes was computed as *mutation count* × (*partition size*_essential_ ÷ *partition size*_nonessential_). Relativized mutation count among essential genes was computed as *mutation count* × (1).

| **Replicate ID** | **Partition** | ***d_N_*/*d_S_*** | **Mutation count** | **Partition size** | **Relativized mutation count** |
| --- | --- | --- | --- | --- | --- |
| MM_1 | Nonessential | 0.759 | 8.14 | 408 | 9.83 |
| MM_3 | Nonessential | 0.177 | 17.31 | 408 | 20.92 |
| MM_4 | Nonessential | 0.266 | 11.07 | 408 | 13.38 |
| MM_6 | Nonessential | 0.456 | 7.68 | 408 | 9.28 |
| MM_1 | Essential | 0.383 | 11.52 | 493 | 11.52 |
| MM_3 | Essential | 0.574 | 16.59 | 493 | 16.59 |
| MM_4 | Essential | 0.383 | 10.67 | 493 | 10.67 |
| MM_6 | Essential | 1.148 | 10.73 | 493 | 10.73 |

**Table S2**. **Deletions in the non-minimal cell.** All genic deletion mutations observed during adaptive evolution across all replicate populations.

| **Strain** | **Replicate ID** | **Loci affected** | **Loci**  **essentiality** | **Putatively adaptive?** | **Deletion size (bp)** | **Mutant allele**  **frequency** |
| --- | --- | --- | --- | --- | --- | --- |
| Non-minimal | MM_1 | *tnpA_1*, *tnpB_1* | Nonessential | Yes | 1483 | 1 |
| Non-minimal | MM_1 | *MMSYN1_0177* | Nonessential | No | 1 | 0.197 |
| Non-minimal | MM_1 | *MMSYN1_0469* | Nonessential | No | 1 | 0.264 |
| Non-minimal | MM_3 | *tnpA_1*, *tnpB_1* | Nonessential | Yes | 1495 | 1 |
| Non-minimal | MM_3 | *MMSYN1_0326* | Essential | No | 1 | 1 |
| Non-minimal | MM_4 | *pepF* | Nonessential | No | 1 | 1 |
| Non-minimal | MM_4 | *MMSYN1_0471* | Nonessential | Yes | 3 | 0.467 |
| Non-minimal | MM_4 | *ftsZ* | Nonessential | Yes | 1 | 1 |
| Non-minimal | MM_6 | *his3*, *tetM*, *lacZ* | Nonessential | Yes | 7047 | 1 |
| Non-minimal | MM_6 | *MMSYN1_0471* | Nonessential | Yes | 1 | 1 |
| Non-minimal | MM_6 | *MMSYN1_0751* | Nonessential | Yes | 1 | 1 |

**Table S3**. ***Mycoplasma* medium.** Recipe for SP4 medium used to culture *Mycoplasma mycoides*. Any use of trade, firm, or product names is for descriptive purposes only and does not imply endorsement by the U.S. Government.

| **Ingredient** | **Manufacturer** | **Quantity/L** | **Unit** |
| --- | --- | --- | --- |
| *Mycoplasma* broth base | Hardy Diagnostics | 3.5 | g |
| Bacto tryptone | Fisher BioReagents | 10 | g |
| Bacto peptone | Fisher BioReagents | 5.3 | g |
| Glucose (20%) | Sigma-Aldrich | 25 | mL |
| Yeastolate (2%) | Life Technologies | 100 | mL |
| Epure filtered water |  | 725 | mL |
| CMRL 1066 (10X) | Corning | 50 | mL |
| 7.5% sodium bicarbonate | Fisher Chemical | 14.6 | mL |
| 200 mM L-glutamine | Fisher Chemical | 5 | mL |
| Yeast extract solution (15%) | Gibco | 35 | mL |
| Knockout serum replacement | Gibco | 170 | mL |
| Penicillin G | Sigma-Aldrich | 100 | μL |
| Phenol red | Sigma-Aldrich | 0.011105 | g |

**Table S4. Genes added to the minimal cell.** Following list of genes were added back to minimized *Mycoplasma mycoides* JCVI-syn3B to improve cell maintenance.

| **Locus Tag** | **Annotation** |
| --- | --- |
| MMSYN1_0520 | Conserved hypothetical protein |
| MMSYN1_0521 | Cell division protein sepF |
| MMSYN1_0522 | Cell division protein ftsZ |
| MMSYN1_0527 | Putative metal binding or nucleic acid binding |
| MMSYN1_0538 | Conserved hypothetical protein |
| MMSYN1_0546 | Pseudogene |
| MMSYN1_0548 | tRNA (cytidine(34)-2'-O)-methyltransferase |
| MMSYN1_0549 | Non-canonical purine NTP pyrophosphatase |
| MMSYN1_0592 | Uncharacterized protein |
| MMSYN1_0593 | Uncharacterized protein |
| MMSYN1_0601 | Putative CAAX amino terminal protease, transmembrane |
| MMSYN1_0602 | Pseudogene |
| MMSYN1_0604 | LemA family protein |
| MMSYN1_0605 | Putative phosphate starvation inducible protein (psiF), |
| MMSYN1_0610 | DNA-formamidopyrimidine glycosylase |
| MMSYN1_0622 | Uncharacterized lipoprotein |
| MMSYN1_0623 | Putative adenine glycosylase, methyltransferase, kinase |
| MMSYN1_0930 | 50S ribosomal protein L33 rpmG |
| MMSYN1_0931 | Adenylyl-sulfate kinase met14p |

**Table S5**. **Growth curve statistics.** Parameters and summary statistics from generalized linear mixed models for lag time yield, which were estimated from growth curves for the non-minimal and minimal cells. The cell type (“Cell”) and time (“Generation”) were treated as fixed effects. We included random intercepts for the replicate populations.

| **Lag time** | |  |  |  |  |
| --- | --- | --- | --- | --- | --- |
| **Fixed effects** |  |  |  |  |  |
|  | **Estimate** | **Standard error** | **df** | ***t*** | ***P*-value** |
| Intercept | 1.26E+01 | 7.41E-01 | 131 | 17.03 | <0.0001 |
| Generation | -3.86E-04 | 3.87E-04 | 131 | -1.00 | 0.321 |
| Cell | -6.79E+00 | 1.04E+00 | 6 | -6.50 | 0.0006 |
| Generation * Cell | 3.05E-04 | 5.38E-04 | 131 | 0.57 | 0.570 |
|  |  |  |  |  |  |
| **Random effects** |  |  |  |  |  |
|  | **Standard deviation** |  |  |  |  |
| Intercept | 1.23 |  |  |  |  |
| Residual | 2.04 |  |  |  |  |
| Observations | 141 |  |  |  |  |
| Groups | 8 |  |  |  |  |
|  |  |  |  |  |  |
| **R^2^** |  |  |  |  |  |
| Marginal | 0.65 |  |  |  |  |
| Conditional | 0.75 |  |  |  |  |
|  |  |  |  |  |  |
| **Variance partition coefficient** | |  |  |  |  |
| VPC | 0.267 |  |  |  |  |

| **Yield** | |  |  |  |  |
| --- | --- | --- | --- | --- | --- |
| **Fixed effects** |  |  |  |  |  |
|  | **Estimate** | **Standard error** | **df** | ***t*** | ***P*-value** |
| Intercept | 2.36E-01 | 1.64E-02 | 126 | 14.03 | <0.0001 |
| Generation | 7.24E-05 | 1.50E-05 | 126 | 4.83 | <0.0001 |
| Cell | 5.60E-01 | 2.24E-02 | 6 | 25.02 | <0.0001 |
| Generation * Cell | -9.63E-05 | 2.06E-05 | 126 | -4.65 | <0.0001 |
|  |  |  |  |  |  |
| **Random effects** |  |  |  |  |  |
|  | **Standard deviation** |  |  |  |  |
| Intercept | 2.13E-06 |  |  |  |  |
| Residual | 6.26E-02 |  |  |  |  |
| Observations | 134 |  |  |  |  |
| Groups | 8 |  |  |  |  |
|  |  |  |  |  |  |
| **R^2^** |  |  |  |  |  |
| Marginal | 0.91 |  |  |  |  |
| Conditional | 0.91 |  |  |  |  |
|  |  |  |  |  |  |
| **Variance partition coefficient** | |  |  |  |  |
| VPC | 6.2E-11 |  |  |  |  |

**REFERENCES**

1. Yus, E. *et al.* Determination of the gene regulatory network of a genome-reduced bacterium highlights alternative regulation independent of transcription factors. *Cell Syst.* **9**, 143-158.e13 (2019).
2. Ferguson, M. & Ihrie, J. MPN: Most Probable Number and other microbial enumeration techniques. R package version 0.3.0. https://CRAN.R-project.org/package=MPN (2019).
