# Supplementary material for: Evolution of a minimal cell
Source: Nature. 2023 Jul 5;620(7972):122–7. doi: 10.1038/s41586-023-06288-x (PMC10396959; doi:10.1038/s41586-023-06288-x)
Supplement: Supplementary file 2 — Reporting Summary [file 41586_2023_6288_MOESM2_ESM.pdf]

nature portfolio

Corresponding author(s): Jay T. Lennon

Last updated by author(s): May 26, 2023

Reporting Summary

Nature Portfolio wishes to improve the reproducibility of the work that we publish. This form provides structure for consistency and transparency in reporting. For further information on Nature Portfolio policies, see our Editorial Policies and the Editorial Policy Checklist.

Statistics

For all statistical analyses, confirm that the following items are present in the figure legend, table legend, main text, or Methods section.

n/a	Confirmed	
☐	☒	The exact sample size (*n*) for each experimental group/condition, given as a discrete number and unit of measurement
☐	☒	A statement on whether measurements were taken from distinct samples or whether the same sample was measured repeatedly
☐	☒	The statistical test(s) used AND whether they are one- or two-sided<br>*Only common tests should be described solely by name; describe more complex techniques in the Methods section.*
☐	☒	A description of all covariates tested
☐	☒	A description of any assumptions or corrections, such as tests of normality and adjustment for multiple comparisons
☐	☒	A full description of the statistical parameters including central tendency (e.g. means) or other basic estimates (e.g. regression coefficient) AND variation (e.g. standard deviation) or associated estimates of uncertainty (e.g. confidence intervals)
☐	☒	For null hypothesis testing, the test statistic (e.g. *F*, *t*, *r*) with confidence intervals, effect sizes, degrees of freedom and *P* value noted<br>*Give P values as exact values whenever suitable.*
☐	☒	For Bayesian analysis, information on the choice of priors and Markov chain Monte Carlo settings
☐	☒	For hierarchical and complex designs, identification of the appropriate level for tests and full reporting of outcomes
☐	☒	Estimates of effect sizes (e.g. Cohen's *d*, Pearson's *r*), indicating how they were calculated

Our web collection on statistics for biologists contains articles on many of the points above.

Software and code

Policy information about availability of computer code

Data collection	Study uses a combination of custom and open-source code that is available via GitHub repository, that will also be archived via Zenodo upon publication.
Data analysis	Analyses were performed using R (4.3.2), vegan (2.6-4), Python (3.10.2), cutadapt (3.4), breseq (0.32.0), ImageJ (1.53), all of which are open-source software platforms. Code available at the following GitHub repository: https://github.com/LennonLab/MinimalCell. Flow cytometry analyses were performed using the following software: NovoExpress (1.6.0), FACSDiva (9), FCS Express (7).

For manuscripts utilizing custom algorithms or software that are central to the research but not yet described in published literature, software must be made available to editors and reviewers. We strongly encourage code deposition in a community repository (e.g. GitHub). See the Nature Portfolio guidelines for submitting code & software for further information.

Data

Policy information about availability of data

All manuscripts must include a data availability statement. This statement should provide the following information, where applicable:

- Accession codes, unique identifiers, or web links for publicly available datasets
- A description of any restrictions on data availability
- For clinical datasets or third party data, please ensure that the statement adheres to our policy

Data are available on GitHub (https://github.com/LennonLab/MinimalCell). Sequences have been have deposited to NCBI SRA as accession PRJNA743406.

Field-specific reporting

Please select the one below that is the best fit for your research. If you are not sure, read the appropriate sections before making your selection.

☒ Life sciences ☐ Behavioural & social sciences ☐ Ecological, evolutionary & environmental sciences

For a reference copy of the document with all sections, see nature.com/documents/nr-reporting-summary-flat.pdf

Life sciences study design

All studies must disclose on these points even when the disclosure is negative.

Sample size	Sample size was determined from historical precedent in similar experiments while achieving a balance between statistical power and logistical feasibility. For the natural selection experiment, the sample size was based on the work of Lenski (2017, PLoS Genet. 13, 4, e1006668 DOI:10.1371/journal.pgen.1006668). The sample size of the mutation accumulation experiment was based on the work of Behringer & Hall (2016, G3 6, 1, 149-160 DOI:10.1534/g3.115.022129).
Data exclusions	Replicate populations or clones were excluded if a heuristic inspection of sequence data suggested that the population or clone from which the data were derived was cross contaminated with another experimental replicate population or clone, or was contaminated with a foreign organism. After inspection, 2 minimal cell natural selection populations, 2 non-minimal cell natural selection populations, 43 minimal cell mutation accumulation clones, and 11 non-minimal cell mutation accumulation clones were removed. When the type of contamination was cross contamination between two experimental replicates, the higher-numbered replicate was chosen to be discarded.
Replication	Several types of observations were tested for the ability to be replicated. Microscopic analyses were replicated using scanning electron microscopy after an initial analysis with phase contrast microscopy. Measurements of evolved fitness were taken with both a competitive fitness metric and a growth rate-based metric. In the flow cytometry analyses, the robustness of the conclusions to alternative gating strategies was verified.
Randomization	Randomization of samples into experimental groups was not applicable to this study because replicate populations of all samples were allocated to all of the experimental groups in separate experiments.
Blinding	To eliminate investigator bias in the mutation accumulation experiment, the colony to be chosen for each transfer was chosen ahead of time, before colonies became visible, by marking the Petri plate with permanent marker. When colonies grew visible, the colony closest to the mark was chosen for the transfer.

Reporting for specific materials, systems and methods

We require information from authors about some types of materials, experimental systems and methods used in many studies. Here, indicate whether each material, system or method listed is relevant to your study. If you are not sure if a list item applies to your research, read the appropriate section before selecting a response.

Materials & experimental systems

n/a	Involved in the study	
☒	☐	Antibodies
☒	☐	Eukaryotic cell lines
☒	☐	Palaeontology and archaeology
☒	☐	Animals and other organisms
☒	☐	Human research participants
☒	☐	Clinical data
☒	☐	Dual use research of concern

Methods

n/a	Involved in the study	
☒	☐	ChIP-seq
☐	☒	Flow cytometry
☒	☐	MRI-based neuroimaging

Flow Cytometry

Plots

Confirm that:

☒ The axis labels state the marker and fluorochrome used (e.g. CD4-FITC).

☒ The axis scales are clearly visible. Include numbers along axes only for bottom left plot of group (a 'group' is an analysis of identical markers).

☒ All plots are contour plots with outliers or pseudocolor plots.

☒ A numerical value for number of cells or percentage (with statistics) is provided.

Methodology

Sample preparation	Cells were fixed with 20 µL of 25% glutaraldehyde and stained with 2X SYBR Green.
Instrument	Novocyte flow cytometer (ACEA Biosciences), LSR II flow cytometer (BD Biosciences)
Software	NovoExpress, FACSDiva, FCS Express
Cell population abundance	Abundances were determined using the NovoExpress software. Abundances were on the order of 1 x 10^8 cells/mL, with typically 1800 - 2700 events per second. Purity was determined through the use of negative controls and axenic controls.
Gating strategy	We used the following cutoffs for registering an event: 453 nm laser B530 (Alias: SYBR Green-H) > 3000 and side scatter (Alias: SSC-H) > 300. Next, boundaries for an mCherry "negative" cell were established by gating axenic mCherry negative populations. Boundaries for an mCherry "positive" cell were established by gating axenic mCherry positive populations. We then used these gates to call each cell "positive" or "negative" in mixed populations. We also obtained the proportion of false negative mCherry cells by using axenic mCherry positive populations; this proportion was then used as a correction factor in mixed populations. We verified the robustness of the conclusions to different gating strategies by redrawing the gates at least once per experiment.

☒ Tick this box to confirm that a figure exemplifying the gating strategy is provided in the Supplementary Information.
